# Epigenetic Regulation of Sialyltransferase ST6GAL1 Drives Breast Tumor Heterogeneity

**DOI:** 10.3390/cancers18182988

**Published:** 2026-09-16

**Authors:** Aparna Maiti, Kei Kawashima, Hongjoo An, Lydia J. B. Redman, Yun Wu, Scott I. Abrams, Joseph T. Y. Lau, Nitai C. Hait

**Affiliations:** 1 Department of Surgical Oncology, Roswell Park Comprehensive Cancer Center, Elm & Carlton Streets, Buffalo, NY 14263, USA; aparna.maiti@roswellpark.org (A.M.);; 2 Department of Gastroenterological Surgery, Yokohama City University Graduate School of Medicine, Yokohama 236-0004, Japan; 3 Department of Breast and Endocrine Surgery, Yokohama City University Graduate School of Medicine, Yokohama 236-0004, Japan; 4 Department of Biomedical Engineering, University at Buffalo, The State University of New York, Buffalo, NY 14260, USA; 5 Department of Immunology, Roswell Park Comprehensive Cancer Center, Elm & Carlton Streets, Buffalo, NY 14263, USA; 6 Department of Molecular & Cellular Biology, Roswell Park Comprehensive Cancer Center, Elm & Carlton Streets, Buffalo, NY 14263, USA

**Keywords:** sialyltransferase ST6GAL1, DNA methylation, epigenetics, breast cancer, metastases, aggressive subtypes, patients’ survival

## Abstract

**Background/Objectives**: Tumor progression is often accompanied by sialylation of cell-surface N-glycan glycoproteins with α2-6-linked sialic acids by the sialyltransferase ST6GAL1. Moreover, a hallmark of human cancers is heterogeneity in ST6GAL1 expression among cancer cells, yet the mechanisms and significance of this variability remain poorly understood. Here, we investigate whether epigenetic changes in ST6GAL1 DNA methylation, which affect intracellular ST6GAL1 and cell-surface α2,6-sialylation of glycoproteins, are associated with the biology of human breast cancer (BCa) cells. **Methods**: We analyzed CpG island methylation across spatially distinct regions of the human ST6GAL1 promoter (P2 and P3) and the associated loss of ST6GAL1 expression in primary human breast tumors compared with adjacent normal tissue controls. Epigenetic changes in ST6GAL1 DNA methylation and patient progression-free survival (PFS) were evaluated. Small-molecule epigenetic inhibitors (5-aza-dC and MS-275) were used to assess ST6GAL1 transcriptional reactivation and its effects on intracellular ST6GAL1, cell-surface α2,6-sialylation, and cell adhesion to collagen I in cultured BCa cell models. **Results**: In estrogen receptor (ER)+ and human epidermal growth factor receptor 2 (HER2)+ primary BCa tissues, ST6GAL1 P3-promoter hypermethylation, but not P2-promoter methylation, significantly correlated with loss of ST6GAL1 expression compared with adjacent normal controls. Higher promoter methylation was significantly associated with improved PFS in ER+ and HER2+ BCa subtypes compared with triple-negative breast cancer (TNBC). ST6GAL1 protein expression and relative transcript levels are higher in adjacent normal controls or human normal breast epithelial cells than in human BCa tissue or cultured cells. In BCa cells, native ST6GAL1 is sensitive to degradation by cycloheximide or MG-132 and even to sialidase treatment. Pharmacological reactivation with 5-aza-dC or MS-275 restored intracellular ST6GAL1 expression and functional cell-surface α2,6-sialylation, thereby enhancing BCa cell adhesion to collagen I. Since ST6GAL1 is also released into the extracellular milieu by cancer cells, and extracellular ST6GAL1 (exoST6) may contribute to cancer cell-surface protein α2,6-sialylation, we also found that 5-aza-dC increased exoST6 levels released in the form of exosome vesicles. **Conclusions**: Our results suggest that ST6GAL1 promoter methylation, together with copy-number alterations, drives loss of expression and phenotypic heterogeneity in human BCa, thereby reducing α2,6-sialylation and correlating with improved PFS in the ER+ and HER2+ subtypes. Demethylating agents restore both endogenous ST6GAL1 expression and exosomal exoST6 release in BCa cells, highlighting this targetable epigenetic axis as a promising prognostic biomarker and therapeutic strategy to improve patient survival in progressive diseases. Future studies will focus on evaluating the functional outcomes of targeting cell-native ST6GAL1 versus the exoST6/sialylation pathways and on defining ST6GAL1’s role in driving disease progression in vivo.

## Simple Summary

Sialyltransferase ST6GAL1 and cell-surface glycoprotein sialylation are associated with tumor progression. While tumor heterogeneity in ST6GAL1 expression and in the α2,6-sialylation of glycoproteins is a hallmark of human cancers, the mechanisms driving this variability remain poorly understood. Here, we investigate whether epigenetic changes in ST6GAL1 DNA methylation, which affect intracellular ST6GAL1 and cell-surface protein sialylation, are associated with human breast cancer (BCa). Multiple spatially distinct promoter regions of ST6GAL1 mediate its transcription. DNA methylation at the ST6GAL1 promoters is tightly associated with the loss of ST6GAL1 expression in primary breast tumors compared with adjacent normal breast tissues and with improved progression-free survival (PFS) in BCa patients. Transcriptional reactivation of ST6GAL1 expression by epigenetic small molecules, e.g., 5-aza-dC or MS-275, resulted in increased intracellular ST6GAL1 expression, cell-surface α2,6-sialylation of glycoproteins, and enhanced adhesion of BCa cells to a collagen I-coated plate. Since ST6GAL1 is also released into the extracellular milieu by cancer cells, and extracellular ST6GAL1 (exoST6) may contribute to cancer cell-surface protein α2,6-sialylation, we also found that 5-aza-dC increased exoST6 levels released in the form of exosome vesicles. Our results suggest that epigenetic regulation of cell-native ST6GAL1 expression and exoST6 release drive heterogeneity in cell-surface glycoprotein sialylation and clinical phenotypes in BCa.

## 1. Introduction

Breast cancer (BCa) is a leading cause of cancer-related mortality among women in the US, estimated at over 42,000 deaths in 2026, with its metastatic progression involving complex multistep processes mediated by genetic, epigenetic, and microenvironmental factors [1,2,3]. Although triple-negative breast cancer (TNBC) is the most aggressive subtype compared with estrogen receptor (ER), progesterone receptor (PR), and human epidermal growth factor receptor 2 (HER2)-positive subtypes, across all subtypes, metastatic progression to distant organs like the bones, lungs, liver, and brain causes over 90% of all BCa patient deaths [4,5]. Aberrant protein glycosylation, particularly hypersialylation, has emerged as a crucial driver of malignant cell behavior and BCa progression and metastasis [6,7,8].

Sialyltransferase ST6GAL1 is a key enzyme natively expressed by cells that resides within the intracellular ER/Golgi secretory network, where it catalyzes the addition of α2-6-linked sialic acids to N-glycans on cell-surface glycoproteins, influencing signal transduction, cellular recognition, cell–cell interactions, and metastatic progression [9,10,11,12,13,14]. Aberrant expression of ST6GAL1 and the resulting alteration in cell-surface sialylation patterns have been linked to cancer progression, metastasis, and resistance to therapy in several malignancies, including BCa. ST6GAL1-mediated sialylation on cell-surface targets, tumor necrosis factor receptor 1 (TNFR1), transforming growth factor receptor beta (TGF-β), epidermal growth factor receptor (EGFR), and integrins, activates downstream signaling, promotes the epithelial-to-mesenchymal transition (EMT), stimulates integrin-mediated tissue invasion, and drives profound resistance to both chemotherapy and radiotherapy [9,10,11,12,13,14]. A major hallmark of human cancers is the marked heterogeneity in ST6GAL1 expression and α2,6-sialylation among tumor cells [15,16], but the underlying mechanisms and clinical relevance of this heterogeneity remain largely unexplored.

Transcriptional regulation of the human ST6GAL1 gene is exceptionally complex and is mediated by several spatially distinct promoter regions (P1, P2, P3, and P4), which generate mRNA isoforms with unique 5′-untranslated regions (5′-UTRs) while encoding an identical polypeptide [17,18,19,20]. ST6GAL1 P1 promoter activity drives hepatocyte-specific expression and circulating soluble ST6GAL1; the P3 promoter is the primary promoter regulating native ST6GAL1 expression across diverse tissues and cancer cells [9]. The genomic and epigenetic changes, particularly copy number variation (CNV) and promoter cytosine-phosphate-guanine (CpG) island DNA methylation, are the drivers of ST6GAL1 regulation in human cancers [21]. Additionally, emerging evidence suggests that reversible epigenetic modifications, particularly promoter DNA methylation and histone modifications, dynamically regulate gene expression in cancer, thereby contributing to the heterogeneity in ST6GAL1/sialylation [22,23]. To date, the DNA methylation status of ST6GAL1 remains largely unexplored, leaving unclear how far ST6GAL1 promoter DNA methylation or the CNV accounts for the loss or heterogeneity of ST6GAL1 across BCa subtypes.

ST6GAL1 and other glycosyltransferases were also found in systemic circulation [24,25,26,27,28,29,30]. While much of the circulating exoST6 originates from the liver, specifically from the P1 Promoter of ST6GAL1, other host cells and cancer cells also contribute to the overall exoST6 pool [25,26,27,31,32]. Work from the Lau laboratory demonstrated that exoST6 can modify cell-surface glycans, even on cells that do not natively express ST6GAL1 [25,26,27,31,32], suggesting that the exoST6 axis contributes to the regulatory sialylation of cell-surface proteins. Changes in circulating exoST6 levels are associated with inflammatory states, other disease conditions, and malignancy [33,34,35,36,37,38,39]. Our earlier published results with others revealed that functional exoST6 can act on target TNBC cells that do not natively express ST6GAL1, enhancing TNBC growth, invasion, EMT, and cancer stemness ex vivo [27,32,40], confirming that extracellular sialylation mechanisms may contribute to cancer cell-surface protein α2,6-sialylation linked to disease progression. However, the mechanisms underlying exoST6/sialylation and its clinical significance in BCa subtypes remain unknown.

In this study, we investigate the genomic and epigenetic drivers of ST6GAL1 expression regulation across BCa subtypes; we analyzed ST6GAL1’s CNV and promoter DNA methylation profiles using The Cancer Genome Atlas-Breast Cancer (TCGA-BRCA) cohort. Here, we systematically evaluate the comparative methylation status of the ST6GAL1 promoter CpG island, its impact on ST6GAL1 transcript levels, and its association with clinical significance and patient survival across BCa subtypes. Furthermore, we have determined how epigenetic alterations in DNA methylation regulate ST6GAL1 gene expression and cell-surface α2,6-sialylation of glycoproteins, both of which are associated with the progression of aggressive BCa. We also demonstrate that epigenetic mechanisms of cell-native ST6GAL1 expression and the release of exoST6 may drive heterogeneity in cell-surface α2,6-sialylation, which is associated with the aggressive clinical phenotypes observed in BCa.

## 2. Materials and Methods

### 2.1. Cells and Reagents

Human breast cancer cell lines, MDA-MB-231 and BT-549 (TNBC), MCF-7 (Luminal A), and BT-474 (Luminal B), and non-tumorigenic MCF10A cells were obtained from ATCC (Manassas, VA, USA) and cultured as previously described [27,41,42,43]. Cells were verified to be mycoplasma-free, passaged within 6 weeks, and plated 24 h before treatment. All experiments were performed during the logarithmic growth phase. MG132, Cycloheximide (CHX), and MS-275 were from Sigma-Aldrich; CMP-sialic acid from Millipore Sigma (MilliporeSigma, Burlington, MA, USA); and *C. perfringens* sialidase C from Roche (Roche USA, Indianapolis, IN, USA). Recombinant rat ST6GAL1 (rST6G) was provided by Dr. Kelley W. Moremen (Univ. of Georgia) [44].

### 2.2. Immunoblotting

Western blotting was performed as described [27,41,42,43,45,46,47]. A total of 25 μg of protein was loaded per lane, separated on 10% SDS-PAGE gels, transferred to nitrocellulose membranes, and processed for Western blot analysis. The following primary antibodies were used: ST6GAL1 goat polyclonal antibody (R&D Systems, Minneapolis, MN, USA; cat. # AF5924), RPLP0 polyclonal antibody (Proteintech, Rosemont, IL, USA), GAPDH polyclonal antibody (Cell Signaling Technology, Danvers, MA, USA), and CD63 and Integrin β1 monoclonal antibodies (Santa Cruz Biotechnology, Santa Cruz, CA, USA). In addition, the following secondary antibodies were used for Western blot analysis: peroxidase-conjugated Affipure goat anti-rabbit IgG (#111-035-045) and goat anti-mouse IgG (#115-035-062) (both from Jackson ImmunoResearch Laboratories, Inc., West Grove, PA, USA), and HRP-conjugated anti-Goat IgG antibody (#HAF109, R&D Systems, Inc., Minneapolis, MN, USA).

### 2.3. SNA Lectin Affinity Assay [48]

For lectin precipitation, cell lysates were incubated overnight with rotation at 4 °C in the presence of 100 μg/mL agarose-conjugated Sambucus nigra lectin (SNA; Vector Laboratories, Newark, CA, USA). The resulting lectin–glycoprotein complexes were isolated by centrifugation, washed with lysis buffer, and eluted by boiling in SDS-PAGE sample buffer. Precipitated proteins were resolved via reducing SDS-PAGE and immunoblotted to assess β1-integrin levels.

### 2.4. SNA Staining [27,48]

To confirm that epigenetic reactivation of ST6GAL1 upregulates cell-surface α2-6 sialic acid levels, cells were stained with SNA. Briefly, cultured BCa cells were incubated with FITC-conjugated SNA (Vector Laboratories, Newark, CA, USA, Cat# B-1305) at a 1:400 dilution for 30 min at 4 °C. DAPI-stained cells (BioLegend, San Diego, CA, USA) and lectin binding were visualized using an EVOS FL fluorescence microscope. Images processed with Fiji/ImageJ (NIH) software (version 1.54f) were assessed, including four fields of view from two biological replicates. SNA-lectin staining was expressed as mean pixel brightness (FITC staining only)/the number of DAPI-stained nuclei per image = mean fluorescence intensity (MFI) per cell [27].

### 2.5. Isolation of Extracellular Vesicles (EVs) by Ultracentrifugation

As previously described [27], EVs were isolated from the BCa cell-conditioned medium. Briefly, the supernatants were collected and centrifuged at 500× *g* for 20 min at 4 °C to discard cells. The supernatants were further centrifuged at 10,000× *g* for 30 min at 4 °C to remove cell debris and apoptotic bodies. Finally, the supernatants were ultracentrifuged at 100,000× *g* for 90 min at 4 °C to precipitate the EVs. The EVs were resuspended in a 20-fold volume of ice-cold buffer (25 mM HEPES, pH 7.0 in 1× PBS) and washed by centrifugation again at 100,000× *g* for 90 min at 4 °C. The washed pellet (1/10th volume of original supernatant) was designated as exosome particles. The protein concentration of the nanoparticles was measured and analyzed by Western blot using ST6GAL1 and an EV-specific marker antibody (CD63) [27].

### 2.6. Immunoprecipitation and rST6 Treatment

For immunoprecipitation (IP), cell lysates [49] were precleared with Protein A/G beads (Invitrogen, Carlsbad, CA, USA), and β1 integrin was immunoprecipitated using the established protocol [41,42] with anti-β1 integrin incubation and followed by Protein A/G beads-mediated capture of protein complexes. Beads were washed extensively and treated with ±rST6G (5 µg/mL) and CMP-Sia (100 µM) for 1 h at 37 °C. Reactions were stopped with excess (10-volume) ice-cold PBS (1×) and processed for Western blot analysis.

### 2.7. Real-Time PCR

Quantitative real-time PCR (qPCR) analysis was performed as described before [27,42,43,50,51,52]. Briefly, total RNA was prepared with TRIzol (Life Technologies, Carlsbad, CA, USA). DNA contamination was removed from RNA with DNase treatment (Qiagen, Germantown, MD, USA). RNA (2 μg) was reverse-transcribed using the High-Capacity cDNA Archive Kit (Life Technologies). cDNAs were diluted 10-fold (target genes) and 100-fold (housekeeping gene GAPDH and amplified with SYBR Green qPCR on a CFX96 cycler (Bio-Rad, Hercules, CA, USA). Gene expression levels were calculated by the 2^−(ΔΔCT)^ method, and for some experiments, the 2^−(ΔCT)^ method [28] was normalized to GAPDH expression. In this manuscript, primers are used for real-time PCR.

Human-ST6GAL1-F: 5′-GCAAAGATCAGAGTGAAACAG-3′, human-ST6GAL1-R: 5′-CACCTCATCGCAGACATG-3′; human-GAPDH-F: 5′-CTTAGCACCCCTGGCCAAG, human-GAPDH-R: 5′-GATGTTCTGGAGAGCCCCG-3; human RPL0-F: 5′-GCAGCATCTACAACCCTGAAG-3′; human RPL0-R: 5′-CACTGGCAACATTGCGGAC-3′; human ST6GAL2-F: 5′-AAGGGGAACGTCTCTTCCAAA-3′; human ST6GAL2-R: 5′-CTTGTTGGCGGTCAGGTAATC-3′. All the validated primer sequences were used from the publicly available Primer Bank (https://pga.mgh.harvard.edu/primerbank/index.html, accessed on 3 August 2026).

### 2.8. Cell Adhesion Assay [18]

Cells (5 × 10^4^) were plated onto 24-well culture plates pretreated with 40 μg/mL bovine collagen I and blocked with 2% tissue culture grade bovine serum albumin (BSA, Sigma, St. Louis, MO, USA). To control for nonspecific binding, cells were also plated onto dishes pretreated with BSA alone. Cells were allowed to adhere for 30 min at 37 °C, and then samples were washed gently with PBS. The remaining adherent cells were fixed with paraformaldehyde (4%), then stained with DAPI, counted, and processed in ImageJ (version 1.54g). The number of cells without the washing procedure was defined as 100%.

### 2.9. BCa Cohort Data Acquisition and Analysis

To perform integrated multi-omics analyses, we used TCGA-BRCA cases with publicly available methylation data (N = 893) [21]. Clinical, transcriptomic, and whole-exome sequencing (WES) data were obtained from cBioPortal for Cancer Genomics [53], while methylation data were downloaded from the UCSC Xena platform for our analyses [54].

Genomic positions of the ST6GAL1 and ST6GAL2 genes and exon structures were obtained from the Ensembl genome browser (http://www.ensembl.org) [55]. Genomic positions and annotations of methylation probes were obtained using the Bioconductor annotation package (Bioconductor version: 3.21) “IlluminaHumanMethylation450kanno.ilmn12.hg19.” Supplementary Figures S1 and S2 were generated using the UCSC Genome Browser (https://genome.ucsc.edu) [56]. All genomic coordinates were based on the hg19 reference genome.

Methylation data were analyzed using β-values [57]. For clinical and survival correlation analyses, patients were stratified into ‘High’ and ‘Low’ groups based on the median methylation level calculated for each specific probe. Correlations between DNA methylation and gene expression were evaluated using methylation β-values and log2-transformed transcript-per-million values [log2(TPM + 1)] [58]. Correlations were assessed using Spearman’s rank correlation test [59].

BCa subtypes were determined by immunohistochemical staining for estrogen receptor (ER) and human epidermal growth factor receptor 2 (HER2), and cases were classified as ER-positive/HER2-negative (ER+/HER2−), HER2-positive (HER2+), or triple-negative breast cancer (TNBC). Among the 741 samples included in the methylation analyses, 416 were ER+/HER2−, 114 were HER2+, 127 were TNBC, and 84 were adjacent normal breast tissue samples.

Metastatic and treatment-related cohorts were also analyzed using GSE58999 (N = 44) [60] and GSE65495 (N = 12) [61], obtained from the Gene Expression Omnibus (GEO) using the GEOquery package in R (version 2.76.0) [62].

### 2.10. Statistical Analysis

The BCa cohort data visualization and statistical analyses were performed using R (version 4.5.2) with Bioconductor (version 3.21) [63]. Associations between variables (e.g., gene expression and mutation/deletion scores) were assessed using the Mann–Whitney U test. The Kaplan–Meier method with the log-rank test was used to compare survival curves between groups.

For ex vivo experiments, quantitative data are presented as mean ± SE of three independent replicates. We used a one-way ANOVA for datasets with multiple group comparisons and Tukey’s post hoc test to control the family-wise error rate. All statistical tests were performed using the GraphPad Prism 8 software. *p* < 0.05 was considered statistically significant.

## 3. Results

### 3.1. Human ST6GAL1 Promoter Predictions and Expressions

**Figure 1:**
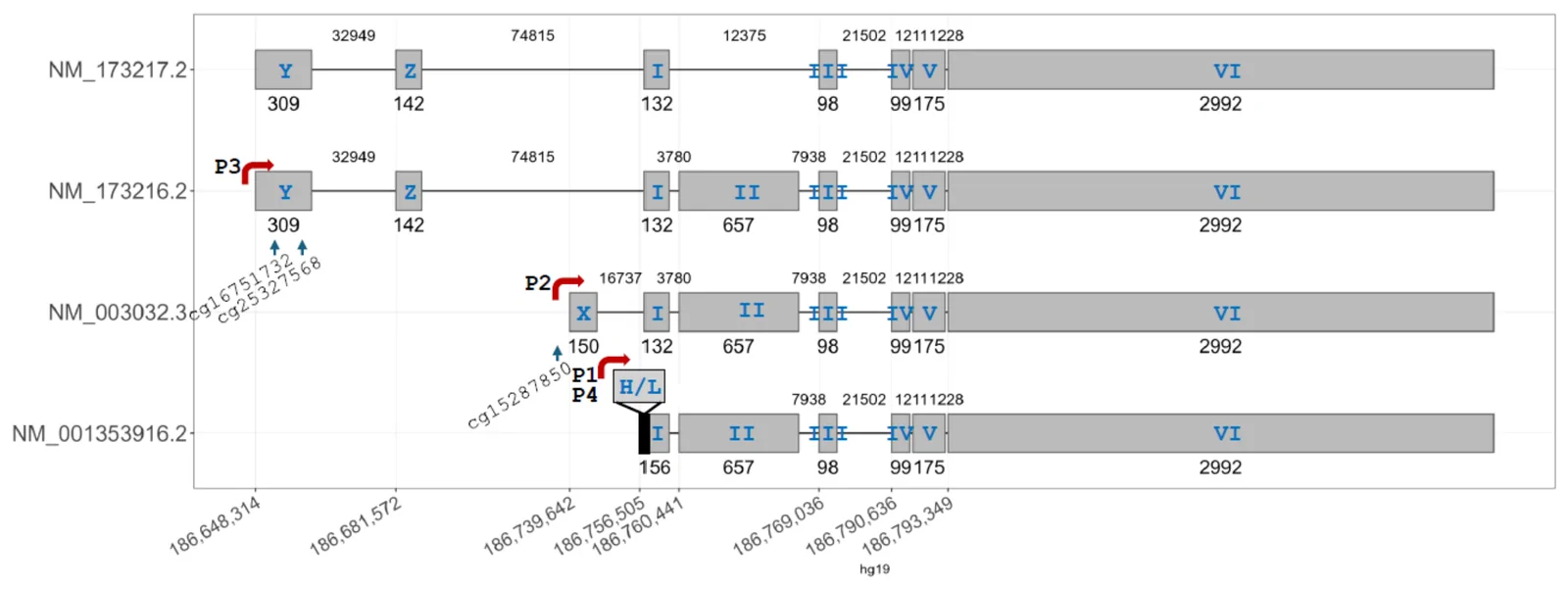
Schematic representation of the genomic organization of the gene encoding ST6GAL1 and of the four major ST6GAL1 transcripts so far identified in human tissues. Genomic organization of the human ST6GAL1 gene (adapted from Dall’Olio [19], 2004; Lo and Lau [64], 1996). The detailed structural layout of the human ST6GAL1 gene was determined using publicly available genomic databases. The four major transcripts from four promoters (*P1*–*P4*) identified in human tissues differ only in their 5′-UTR sequences, reflecting alternative promoter usage. All four transcripts share identical coding exons (I–VI) and therefore encode the same functional ST6GAL1 protein [66].

To identify potential genomic regions for epigenetic changes, such as DNA methylation, we analyzed the genomic sequences extending 145 kb plus covering the transcription start sites of the four predicted ST6GAL1 promoters using available NCBI RefSeq mRNA accession numbers (Figure 1; Dall’Olio et al. [19], 2004; Lo and Lau, J.T [64], 1996). ST6GAL1 features a highly complex transcriptional regulatory mechanism. Tissue- and cell-type-specific ST6GAL1 transcripts are controlled by four known promoters: P1 (exon H, liver, and select cancers), P2 (exon X, B lymphocytes), P3 (exons Y and Z, ubiquitous, cancers), and P4 (exon L, mammary gland). All four promoters produce the same functional ST6GAL1 protein. Full-length functional human ST6GAL1 (P1–P4 promoters: 47–50 kDa ST6GAL1 protein) is a type II membrane glycoprotein localized to the trans-Golgi network, having a catalytic domain that contains two consensus N-glycosylation acceptor motifs located at residues Asn-146 and Asn-158. In contrast to the full-length ST6GAL1 enzyme, the soluble ST6GAL1 protein (liver-specific P1 promoter: 39–44 kDa ST6GAL1) is synthesized in hepatocytes and begins as a full-length membrane-bound enzyme; its passage through the trans-Golgi network subjects it to endoproteolytic cleavage by the aspartyl protease BACE1 (beta-site APP cleaving enzyme 1), which produces a secreted functional exoST6 in human serum [9,24,31,65]. Full-length ST6GAL1 is also released into the extracellular medium as exosomal vesicles, forming functional exoST6 [25,26,27,31,32,40].

To evaluate the epigenetic landscape of the human ST6GAL1 locus, we analyzed upstream genomic sequences from the Ensembl database and identified CpG-rich clusters using the MethPrimer software (v1.0) suite [67]. We found five CpG islands mapped within the human ST6GAL1 promoters P3 and P2, but not at the P1 promoter (Supplementary Figure S1). We found four human ST6GAL1 CpG loci at the non-coding exon Y (P3 promoter) and one CpG locus on the non-coding exon X (P2 promoter). The only other sialyltransferase in the ST6GAL family with similar acceptor specificity is ST6GAL2, but ST6GAL2 expression is restricted to neuronal tissues and is not actively expressed elsewhere [68]. We also found multiple CpG island loci (five) upstream of the transcription start site (TSS) of the ST6GAL2 gene (Supplementary Figure S2), and the gene is not expressed in human breast tissues. The precise identity and role of the specific transcripts of ST6GAL1 and exoST6 in BCa progression and metastasis remain to be fully explored.

### 3.2. Human ST6GAL1 Promoter Methylation Is Tightly Associated with ST6GAL1 Expression Loss in Primary Breast Tumor Subtypes, but Not with Normal Breast Tissue Controls

To explore the regulatory expression of human ST6GAL1, we analyzed the associations between ST6GAL1 mRNA expression and CNV as genomic drivers and DNA methylation levels as epigenetic drivers in the TCGA database [21]. In contrast to other human cancers [69], ST6GAL1 expression in BCa is highly sensitive to physical changes in DNA (Supplementary Figure S3A,B). While tumors delete a copy number, ST6GAL1 mRNA expression drops significantly below baseline (non-amplified); conversely, when tumors gain copies, ST6GAL1 mRNA always increases (Supplementary Figure S3A). Furthermore, TNBC tumors have profound copy number deletions compared with ER+/HER2− or HER2+ tumors linked to ST6GAL1 loss, and copy number is amplified compared with baseline in HER2+ but not in ER+/HER2− or TNBC tumors; however, all tumor subtypes showed significant differences between deletion vs. gain of copies (Supplementary Figure S3A,B). Further CNV analysis revealed that TNBC tumors have the highest frequency of ST6GAL1 genomic amplification compared to hormone receptor (HR)-positive breast tumors, suggesting genomic gain is one of the mechanisms driving ST6GAL1 upregulation in aggressive breast cancers (Supplementary Figure S3A,B) [12,27,70].

**Figure 2:**
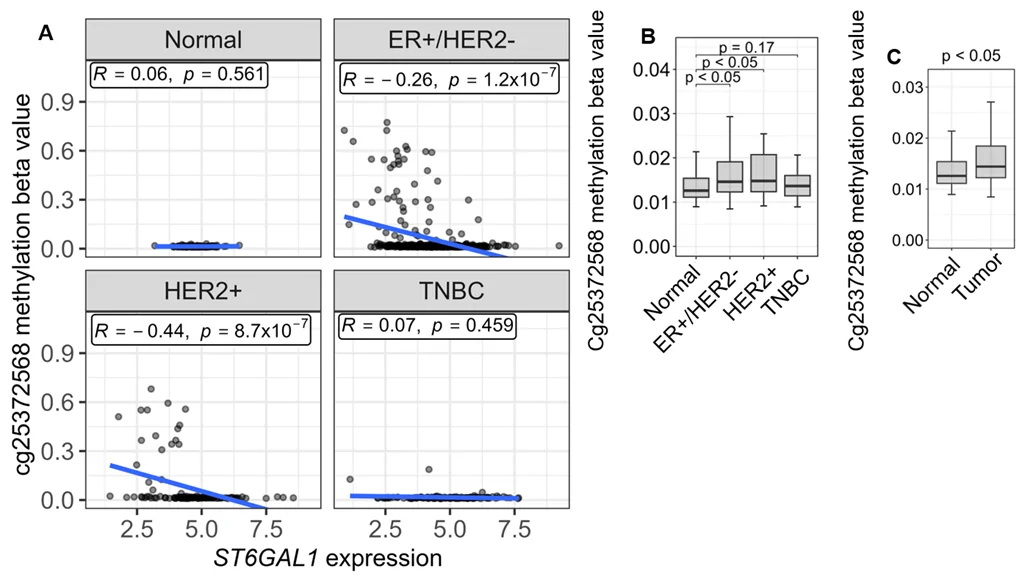
Tumor-specific ST6GAL1 promoter hypermethylation is associated with *ST6GAL1* gene silencing in an independent TCGA-BRCA dataset with the cg25372568 DNA methylation locus. (**A**) An inverse correlation between ST6GAL1 mRNA expression and its DNA methylation status at the cg25372568 methylation probe in primary BCa samples (TCGA-BRCA cohort) in ER+/HER2− or HER2+ subtypes, but not in TNBC or adjacent control breast samples. ρ: Spearman correlation coefficient. (**B**) Subtyping of BCa patients was based on IHC-assessed ER/HER2 status from the TCGA-BRCA clinical data. DNA methylation (cg25372568) levels in various BCa subtypes were compared with adjacent control breast tissue samples. (**C**) Comparison of methylation levels between cancer and normal breast tissue controls.

**Figure 3:**
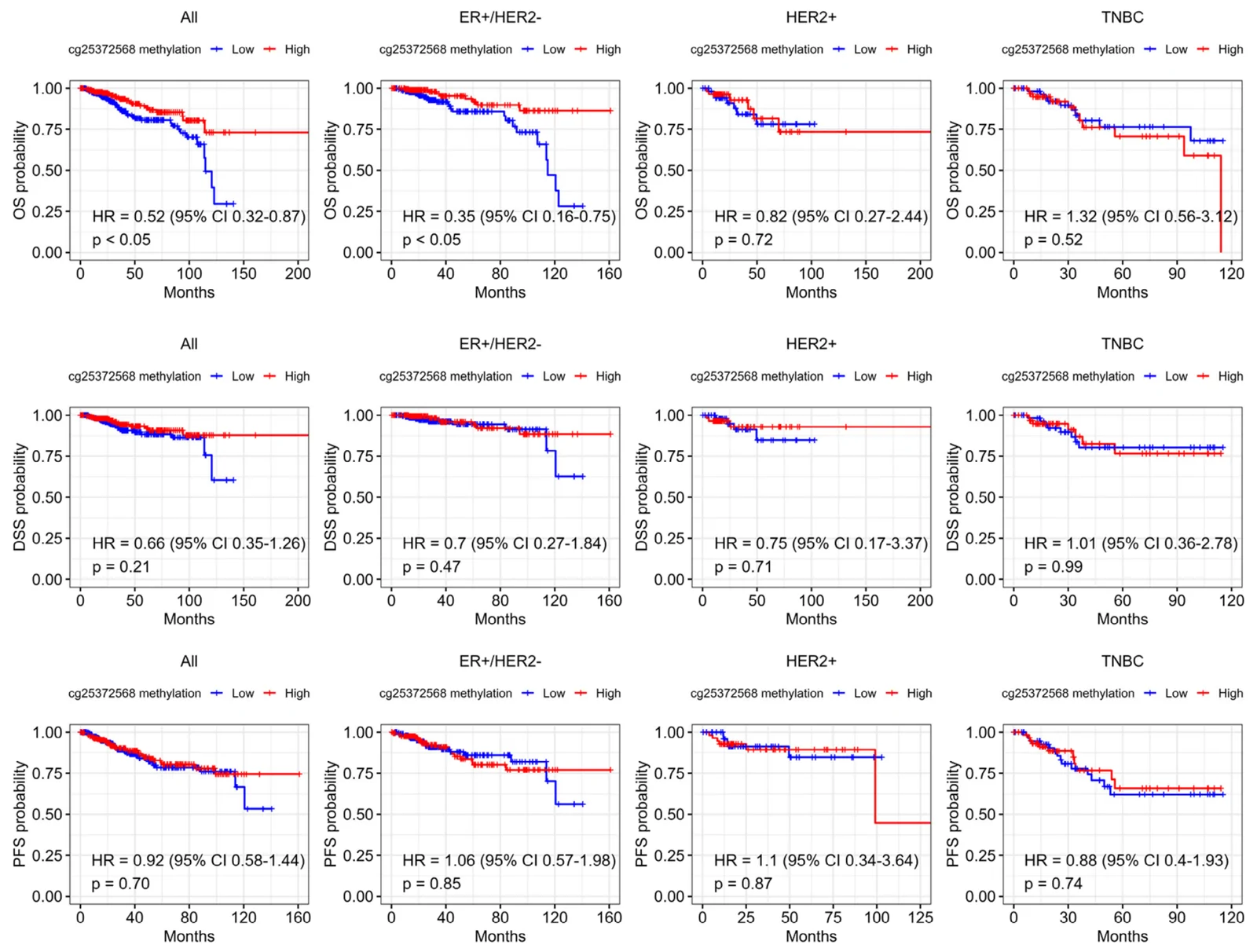
Tumor-specific ST6GAL1 promoter hypermethylation is associated with ST6GAL1 gene silencing and patient survival in the TCGA-BRCA dataset, as indicated by cg25372568. Methylation data for the probe were analyzed using β-values. For comparisons of patient survival outcomes, cases were dichotomized into high and low groups based on the median methylation value per probe. OS: overall survival; DSS: disease-specific survival; PFS: progression-free survival.

We examined human ST6GAL1 mRNA expression and its promoter DNA methylation using TCGA-BRCA genomic data. Analysis of the ST6GAL1 P3 promoter locus reveals multiple promoter regions with variable CpG content. Our data analysis identified a critical CpG site, cg25372568 [17], located precisely within the ST6GAL1 P3 promoter region (+230 base pairs relative to the transcription start site on chr3:186,648,544). When analyzing primary human breast tumor tissues across various molecular subtypes, the methylation status of the cg25372568 locus varied significantly and demonstrated a strong inverse correlation with transcription (Figure 2A). The methylation of the ST6GAL1 promoter was higher in tumor tissues than in normal breast tissue controls. The promoter methylation levels (cg25372568 locus) were lower in normal adjacent primary breast tissues compared with the BCa subtypes, including ER+/HER2− and HER2+, but not in the TNBC subtype (Figure 2B,C). We further analyzed the prognostic significance of ST6GAL1 methylation in BCa patients, and our results showed that all BCa patients with high ST6GAL1 methylation (cg25372568 probe) had a more favorable overall survival (OS) or disease-specific survival (DSS) than those with low ST6GAL1 methylation (Figure 3). Interestingly, these patient survival data were also significant in ER+ or HER2+ BCa patients, but not in TNBC patients. These results suggested that the cg25372568 locus of the ST6GAL1 P3 promoter was linked to higher promoter methylation and ST6GAL1 loss of expression, and that this association was at least observed in ER+ or HER2+ patients.

**Figure 4:**
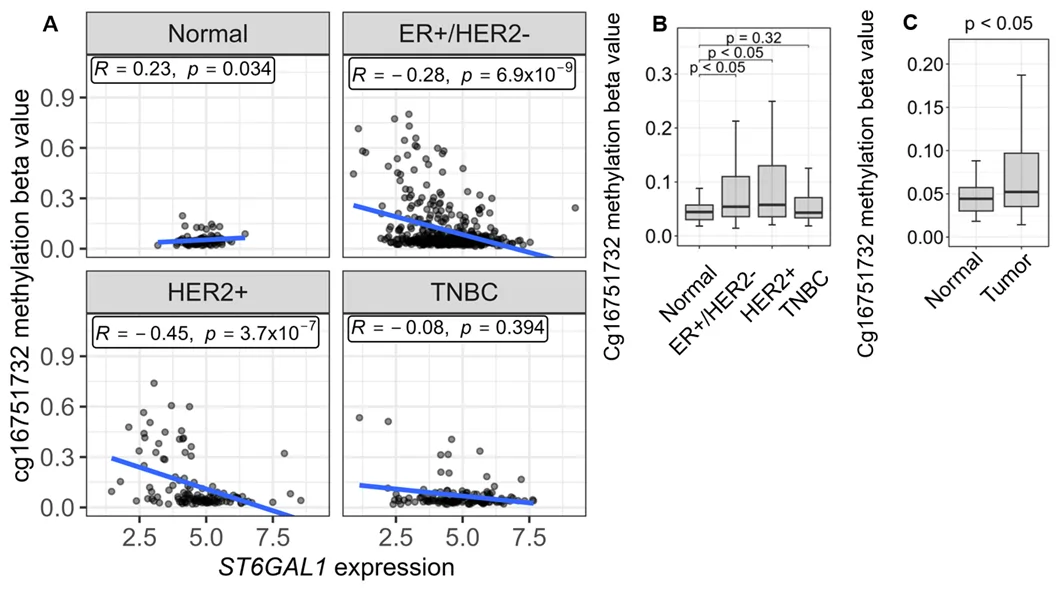
Tumor-specific ST6GAL1 promoter hypermethylation is associated with ST6GAL1 gene silencing in an independent TCGA dataset, as indicated by cg16751732. (**A**) An inverse correlation between *ST6GAL1* mRNA expression and its DNA methylation status at the cg16751732 methylation probe in primary BCa samples (TCGA-BRCA cohort) in ER+/HER2− or HER2+ subtypes, but not in TNBC or adjacent control breast samples. ρ: Spearman correlation coefficient. (**B**) Subtyping of BCa patients was based on IHC-assessed ER/HER2 status from the TCGA-BRCA clinical data. DNA methylation (cg16751732) levels in various BCa subtypes were compared with adjacent control breast tissue samples. (**C**) Comparison of methylation levels between cancer and normal breast tissue controls.

To evaluate ST6GAL1 promoter methylation, we also analyzed the methylation status of another locus (cg16751732) [69], the P3 promoter of the human ST6GAL1 (+78 base pairs relative to the transcription start site on chr3:186,648,392). As expected, promoter methylation (cg16751732 locus) levels are higher in cancer vs. normal adjacent primary tumor tissues, and methylation levels are inversely correlated with ST6GAL1 expression in ER+/HER2- or HER2+ BCa patients but not in TNBC patients (Figure 4A–C). However, Supplementary Figure S4 data suggested that methylation at the cg16751732 locus is not associated with BCa patient survival.

**Figure 5:**
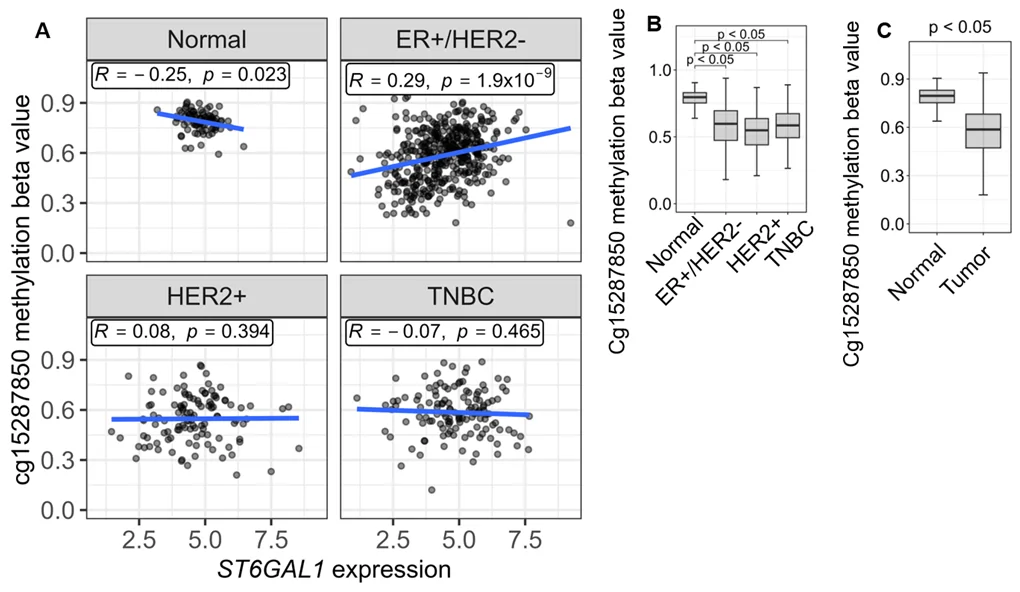
Tumor-specific ST6GAL1 promoter hypermethylation is associated with ST6GAL1 gene silencing in an independent TCGA dataset, as indicated by cg15287850. (**A**) An inverse correlation between ST6GAL1 mRNA expression and its DNA methylation status at the cg15287850 methylation probe in primary BCa samples (TCGA-BRCA cohort) in ER+/HER2− or HER2+ subtypes, but not in TNBC or adjacent control breast samples. ρ: Spearman correlation coefficient. (**B**) Subtyping of BCa patients was based on IHC-assessed ER/HER2 status from the TCGA-BRCA clinical data. DNA methylation (cg15287850) levels in various BCa subtypes were compared with adjacent control breast tissue samples. (**C**) Comparison of methylation levels between cancer and normal breast tissue controls.

**Figure 6:**
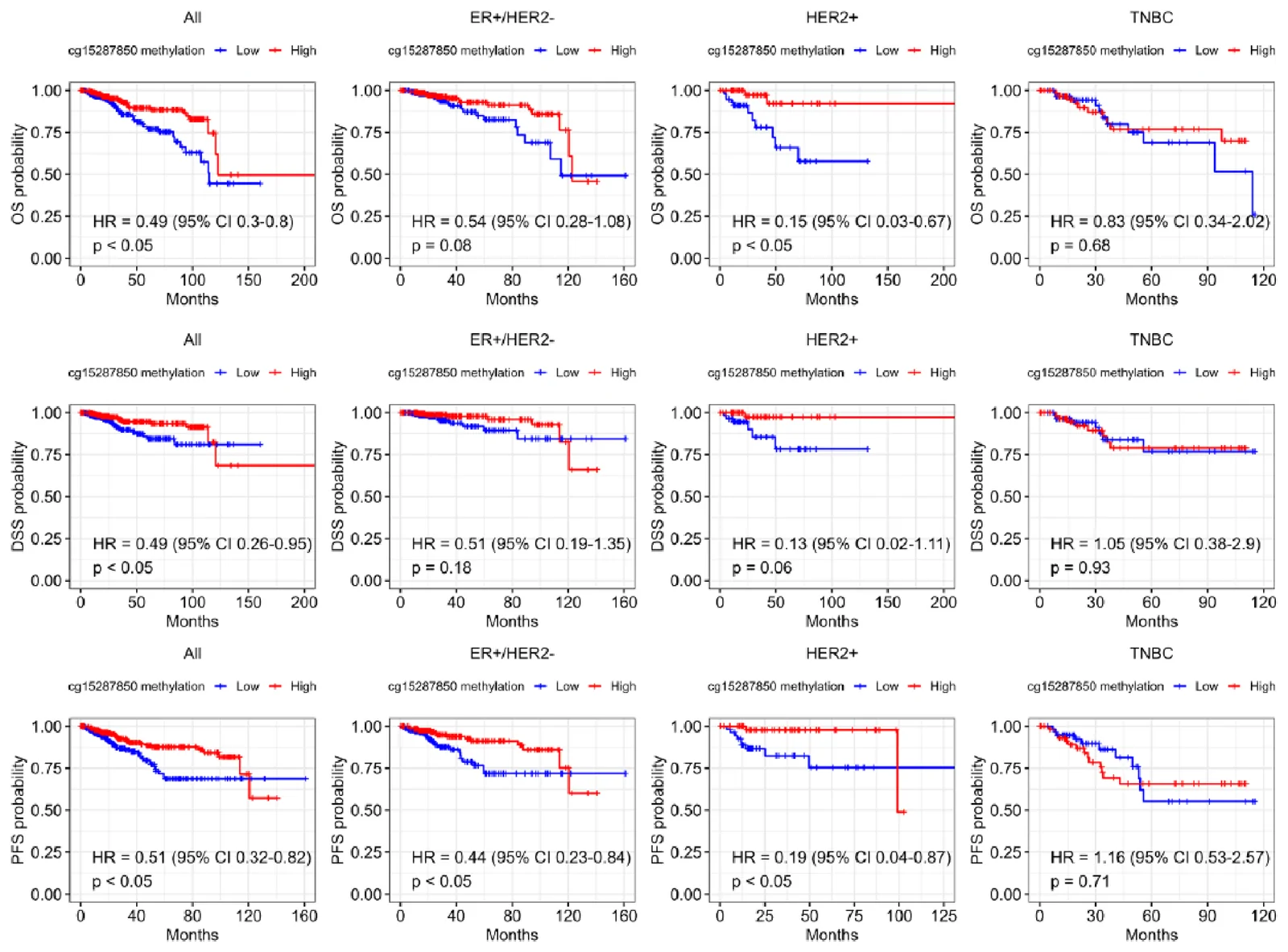
Tumor-specific ST6GAL1 promoter hypermethylation is associated with ST6GAL1 gene silencing and patient survival in the TCGA-BRCA dataset, as indicated by cg15287850. Methylation data for the probe were analyzed using β-values. For comparisons of patient survival outcomes, cases were dichotomized into high and low groups based on the median methylation value per probe. OS: overall survival; DSS: disease-specific survival; PFS: progression-free survival.

Furthermore, we also examined the methylation status of the ST6GAL1 P2 promoter at the cg15287850 locus [71,72] (chr3: 186,739,599, −43 bp os TSS), which represents a clinically significant prognostic marker strongly associated with long-term overall survival in BCa cohorts [71]. Our data revealed that promoter methylation at this locus is higher in adjacent control breast tissues than in BCa patients, including ER+, HER2+, and TNBC patients (Figure 5A–C). However, higher levels of promoter DNA methylation at this locus (cg15287850) were associated with favorable survival (OS, DSS, or PFS) in BCa patients, including ER+ or HER2+ cases, but not in TNBC patients (Figure 6). Together, these data suggest that human ST6GAL1 expression is regulated by complex promoter mechanisms, mediated by DNA methylation, in cancer patients (summarized in Supplementary Table S1).

ST6GAL1 promoter methylation and ST6GAL1 protein expression scores were used to assess associations with key clinopathological features, including age, sex, race, menopausal status, histological grade, stage, and IHC subtyping (Supplementary Tables S2–S5). Data revealed that ST6GAL1 P3-promoter methylation (cg25372568 and cg16751732) is associated with BCa IHC subtyping (*p* < 0.05). In contrast, methylation at the cg16751732 locus is associated with pre- and post-menopausal status in BC patients (*p* < 0.05), and cg15287850 methylation scores are associated with race and grade in BCa patients (Supplementary Tables S2–S4). Further, we found significant associations between the ST6GAL1 protein expression score and IHC subtyping and menopausal status in BC patients (Supplementary Table S5). BCa patients with lymph node (LN) metastases are known to have a higher likelihood of developing distant metastases [73]. Although ST6GAL1 mRNA expression levels are reduced in a small number of metastatic patients (N = 7) in the TCGA-BRCA patient cohort (Supplementary Figure S5A), data analysis of the other BCa LN metastasis cohort (GSE58999) [60] suggested that DNA methylation levels at either the ST6GAL1 P3 or P2 promoter loci did not differ significantly between primary and LN metastatic sites (Supplementary Figure S5B–D).

### 3.3. ST6GAL1 Protein Levels Are Reduced in Cancers

**Figure 7:**
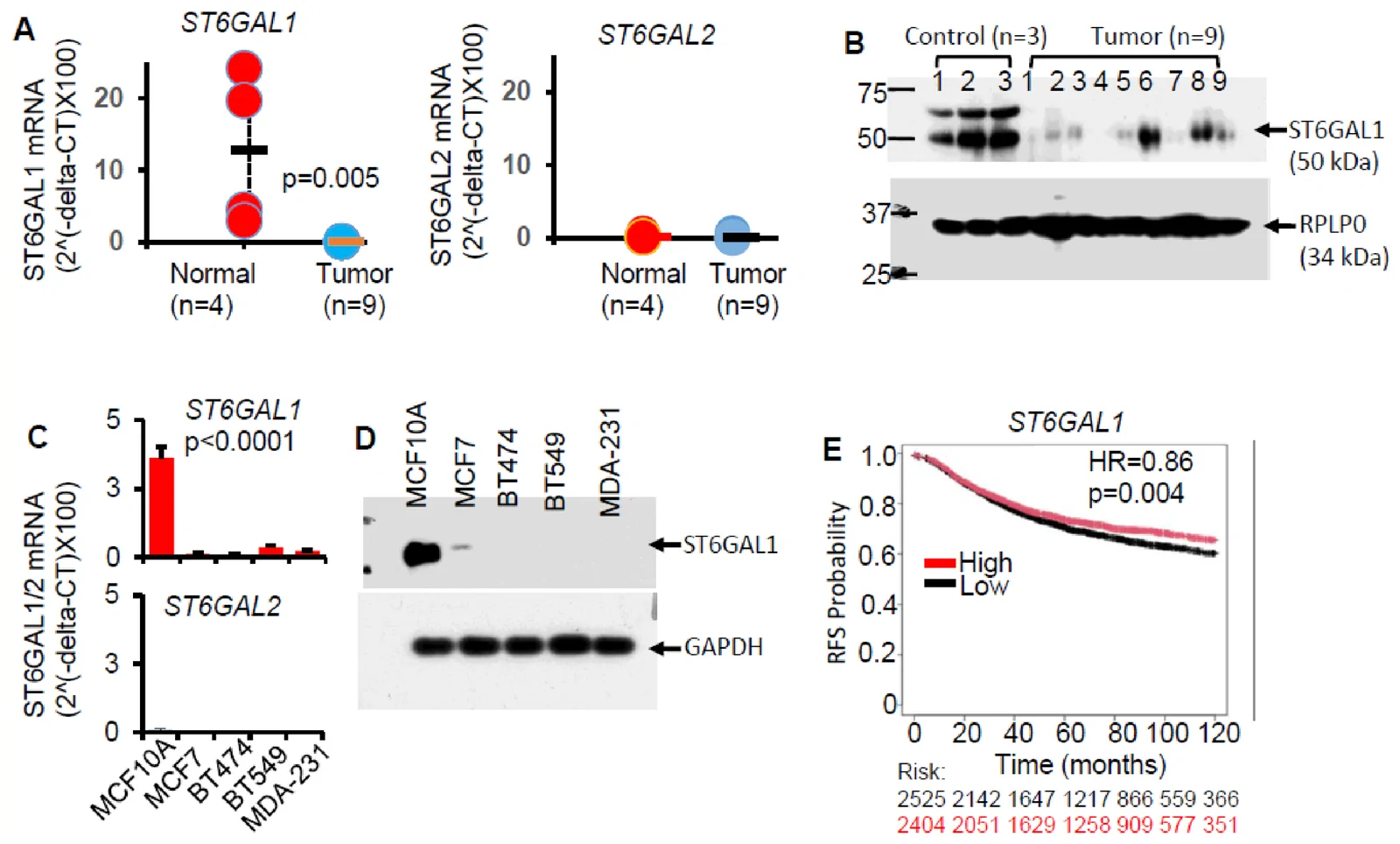
ST6GAL1 expression levels are suppressed in BCa patient samples. (**A**) Tissue samples from ER+ (tumors 1–3) and TNBC (tumors 4–9) patient-derived xenografts (PDX) [42] and normal breast tissue controls (cont. breast 1–3) were used for quantitative real-time PCR. mRNA levels of ST6GAL1 and ST6GAL2 were normalized to RPLP0 and calculated by the Delta CT method. Data are means ± SD, *p* < 0.05 vs. control breast, by Student’s *t*-test. (**B**) Equal amounts of tissue lysates from (**A**) were subjected to Western blot analyses with specific antibodies against ST6GAL1 and RPLP0, with a loading control as indicated. N = 3; representative blots are shown. (**C**,**D**) Human breast epithelial MCF10A, ER+ (MCF-7 and BT474), and TNBC (BT549 and MDA-MB-231) cells were used to analyze ST6GAL1 expression levels. (**C**) ST6GAL1 and ST6GAL1 mRNA levels were normalized to the housekeeping gene GAPDH and calculated using the Delta CT method. Data are expressed as means ± SD. ANOVA and Tukey’s ad hoc analyses, *p* < 0.05 vs. MCF10A normal epithelial cell control. (**D**) Duplicate cell lysates were used for ST6GAL1 Western blot analysis; GAPDH was used as an equal loading and transfer control. N = 3; representative blots are shown. (**E**) The Kaplan–Meier method estimates the probability of relapse-free survival (RFS) in BCa patients by mRNA. Analysis was performed using the online KM-plotter [74].

Next, we examined ST6GAL1 protein expression in a cohort of human BCa patient-derived xenografts (PDXs) and in adjacent normal control breast tissue specimens. As expected, *ST6GAL1* mRNA levels were markedly reduced in tumors compared with normal breast tissue controls, and ST6GAL1 protein levels were higher in normal tissues than in tumors (Figure 7A,B). The other variant, ST6GAL2, is mainly expressed in the human brain, and transcript levels were barely detectable in tumors or adjacent normal breast tissue specimens (Figure 7A). Similarly, we found that ST6GAL1 transcript and protein were predominantly expressed in normal human breast epithelial cells compared with human BCa cells of various subtypes, including luminal A (MCF7) and luminal B (BT474), as well as TNBC (MDA231 and BT549) (Figure 7C,D). ST6GAL2 mRNA levels were almost undetectable in those cells. Surprisingly, our KM plotter cohort analysis [74] of all BCa patients revealed that lower ST6GAL1 transcript levels were associated with unfavorable outcomes, including relapse-free survival (RFS) (Figure 7E). Together, the data suggested that ST6GAL1 expression is regulated in a complex manner in BCa patients and cultured cells, and that promoter methylation may be a key mechanism underlying this regulation.

### 3.4. Epigenetic Reactivation of ST6GAL1 and Release of exoST6 in BCa Cells

**Figure 8:**
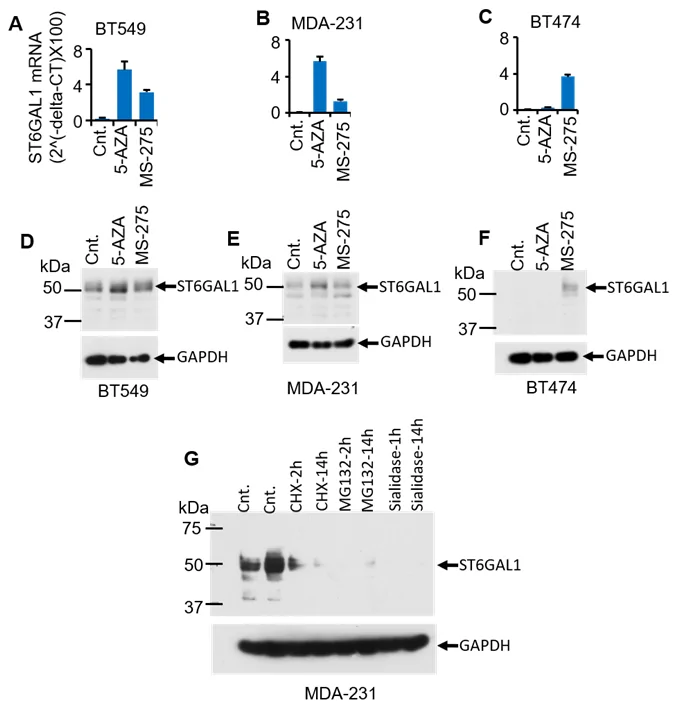
ST6GAL1 expression analysis in 5-aza-dC and MS-275-treated BCa cells. (**A**–**C**) qPCR and (**D**–**F**) Western blot analysis of ST6GAL1, (**A**,**D**) BT-549, (**B**,**E**) MDA-MB-231, and (**C**,**F**) BT-474 cells treated with 5 µM of 5-aza-DC and 1 µM of MS-275 for 24 h. (**A**–**C**) Total RNA was isolated separately from individual cells and treated with DNase. cDNA was prepared from total RNA and used for SYBR-Green qPCR analysis of ST6GAL1 transcript expression. GAPDH mRNA expression was used to normalize ST6GAL1 transcript levels. Normalized transcript expression levels were calculated using the Delta CT method (N = 3); data are means ± s.d. One-way ANOVA (*p* < 0.001), Student’s *t*-test, and *p* < 0.001 (drug-treated cells vs. vehicle control cells). (**D**–**F**) Total cell lysates (15 µg) from the duplicate experiment were used for Western blot analysis with antibodies against ST6GAL1. GAPDH was used to show equal loading and transfer. N = 3; representative blots are shown. (**G**) *Cell-native ST6GAL1 is a glycoprotein and structurally unstable.* MDA-MB-231 cells were treated with vehicle control, 60 µg/mL cycloheximide (CHX) [81] or 0.5 µM MG132, or with *C. perfringens* sialidase C (Roche; 20 µL/mL) [25], as indicated, and cell lysates (45 µg/sample) were used for Western blot analysis of ST6GAL1. GAPDH was used as a loading control. N = 3; representative blots are shown. Note: similar data were obtained using BT549 and BT-474 cells.

To confirm that ST6GAL1-promoter hypermethylation directly represses its transcription, BCa cell lines were treated with chromatin-remodeling drugs. Treatment with a potent DNA methyltransferase (DNMT) inhibitor and DNA hypomethylating agent (5-aza-dC) or a selective class I histone deacetylase (HDAC) inhibitor (MS-275) successfully reversed epigenetic silencing [75,76], yielding a robust, transcript-specific increase in cellular ST6GAL1 mRNA and protein expression levels in cultured BCa cells (Figure 8A–F). In agreement, data analysis using a patient-derived xenograft (PDX) treatment cohort (hypomethylating agent, Decitabine, GSE171073, N = 4) [77] revealed a trend toward reduced ST6GAL1 P3-promoter methylation and a trend toward increased ST6GAL1 transcripts (Supplementary Figure S6A,B). Furthermore, BCa treatment cohort data analysis (GSE65495) [61] revealed a trend of elevated ST6GAL1 transcripts with the selective class I/IV HDAC inhibitor (MGCD) (Supplementary Figure S6C). Next, we specifically investigated the intracellular stability of ST6GAL1in cultured human BCa cells, including TNBC cells. N-linked self-sialylation at the Asn-146 and Asn-158 sites is critical for human ST6GAL1’s structural stability [78,79]. Sialidase treatment removes cellular protein/lipid conjugated sialic acid [25] and also destabilizes and promotes proteasomal degradation of cellular ST6GAL1 protein levels in cultured BCa cells (Figure 8G).

**Figure 9:**
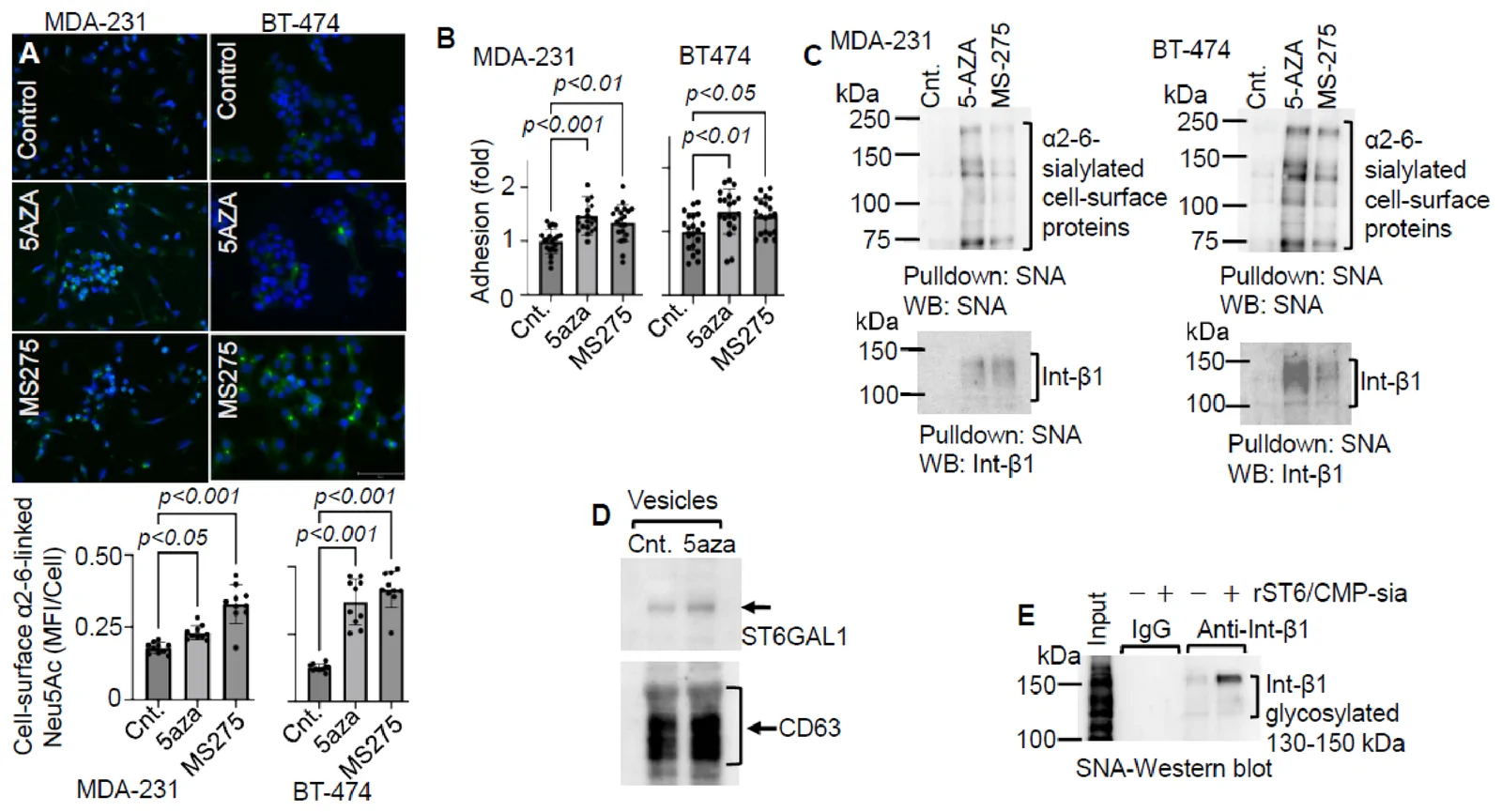
ST6GAL1 is released extracellularly in exosomes in 5-aza-dC-treated BCa cells. (**A**) Immunohistochemical analysis of cell-surface SNA lectin. 5-aza-dC or MS275-treated BCa cells were immunostained with FITC-labeled SNA lectin and DAPI (nuclei). During fluorescence microscopy, exposure times and weighting for both DAPI and FITC fluorescence were kept consistent across samples. (**A**), (bottom panels): Representative images (N = 10) are shown on a scale bar of 50 µm. Images were processed in ImageJ (version 1.54f); background subtraction and MFI per cell calculations were carried out using the same parameters for each condition, and mean fluorescence intensity (MFI) per cell was shown as mean ± s.d. for 10 fields of view. (**B**) In vitro BCa cell adhesion assay: 24-well plates coated with collagen I (Sigma, USA) were used to evaluate relative cell adhesion of BCa cells treated with 5-aza-dC and MS275. Compared with the vehicle-treated control, the 5-aza-dC- or MS275-treated cells showed a significant increase in cell adhesion to collagen I. Data are expressed as means ± SE. Values of 20 from a representative experiment; ANOVA and Tukey’s ad hoc analyses, *p* < 0.05 vs. vehicle control. (**C**) Cell-surface protein sialylation and β1-integrin sialylation analysis: Control (cntl), 5-aza-dC, or MS275-treated BCa cells (MDA-231 and BT474) were lysed in RIPA+ buffer, and extracted proteins were diluted (>10-fold) in low-salt buffer with protease inhibitor cocktail (1×). A total of 500 µg protein from each cell extract was pulled down with an equal amount of SNA-agarose beads. Sialylated protein complexed SNA beads were washed extensively and processed for Western blot analysis (SNA, top panels; β1integrin, bottom panels). Note: equal amounts of protein in the input samples were validated by β-tubulin Western blotting, and control agarose beads were used as SNA pulldown controls; no sialylated protein bands were detected by Western blotting. (**D**) ST6GAL1 in exosome vesicles in 5aza-dc-treated BCa cells: Equal amounts of protein from exosome vesicles isolated from cell medium (10 million cells grown in 10 mL full-serum medium with 5 µM 5aza-dc or vehicle control for 24 h) [27,32] were used for Western blotting with antibodies against ST6GAL1 (exosomal protein) and CD63 (exosomal marker). (**E**) β1-integrin sialylation by extracellular ST6GAL1 (exoST6): 500 µg total proteins (MDA-231) were immunoprecipitated (IP) with anti- β1-integrin or control IgG and Protein A/G agarose beads. Washed beads were incubated ± recombinant ST6GAL1 (rST6, 5 µg/mL) and CMP-sia (100 µM) for 1 h at 37 °C. Reactions were stopped with an excess volume of buffer (10-fold), and samples were analyzed by Western blotting with SNA-biotin lectin. Specific β1-integrin IP was validated by Western blotting. Exo-ST6-mediated sialylated β1-integrin bands (130–150 kDa) are shown. All Western blots were N = 3; representative blots are shown. All experiments were performed in MDA-231, BT-474, or BT-549 cells, or using protein extracts, and similar data were obtained. Uncropped original Western blot images in Figure S7.

It appears that cellular ST6GAL1 in BC cells is unstable; protein synthesis inhibitors (Cycloheximide, CHX) or proteasome inhibitors (MG132) degrade ST6GAL1 in multiple BCa cell lines (Figure 8G). Together, these data suggest that epigenetic mechanisms are among those involved in ST6GAL1 loss and heterogeneity in BCa cells. Immunohistochemical analysis using FITC-conjugated SNA lectin, which recognizes terminal Gal- or GalNAc-linked α2,6-linked sialic acid [80], demonstrated that treatment of BCa cells with 5-aza-dC or MS275 resulted in the robust de novo expression of cell-surface α2,6-linked sialoglycoconjugates (Figure 9A). This treatment also resulted in a significant increase in adhesion to Collagen I in cultured BCa cells (Figure 9B). Epigenetic reactivation small molecules (5-aza-dC and MS-275) also enhance α2,6-linked sialylation of cell-surface glycoproteins, such as β-integrin, in cultured BCa cells (Figure 9C).

Since functional ST6GAL1 protein is also released into the extracellular milieu in the form of exosome vesicles by the source cells [25,27,32], we found that treatment of BCa cells with 5-aza-dC increased exoST6 release via exosomal vesicles (Figure 9D). Furthermore, functional exoST6 [25,27,32] also profoundly sialylates cell-surface glycoproteins, such as β-integrin, in a non-cellular extracellular manner (Figure 9E).

## 4. Discussion

Understanding the molecular mechanisms underlying heterogeneity in tumor cell ST6GAL1 expression and its impact on cell-surface heterogeneous α2-6 sialylation of glycoproteins is critical for identifying functional biomarkers and novel therapeutic targets in BCa progression and metastasis. Here, we demonstrate that genomic and epigenetic mechanisms reduce ST6GAL1 expression in breast tumors compared with higher levels in adjacent normal breast tissue controls by (1) irreversible copy number deletions in aggressive HER2+ and TNBC subtypes, and (2) reversible epigenetic silencing driven by ST6GAL1-promoter hypermethylation. Interestingly, we found that ST6GAL1 promoter hypermethylation and ST6GAL1 loss are associated with improved PFS in patients with BCa, suggesting a potential protective effect of ST6GAL1 loss in the primary tumor in certain BCa subtypes, such as ER+ or HER2+. Tumor cells also release functional ST6GAL1 into the extracellular milieu; this exoST6 can sialylate cell-surface glycoproteins. Interestingly, epigenetic reactivation of tumor ST6GAL1 with the pharmacological inhibition of DNMT (5-aza-dC) and HDAC (entinostat) not only restored cellular ST6GAL1 expression but also increased exoST6 release, highlighting the plasticity of tumor sialylation patterns. Together, our findings indicate that epigenetic modulation of ST6GAL1 promoter methylation plays a crucial role in regulating ST6GAL1 expression and contributes to heterogeneity in cell-surface glycoprotein sialylation.

Complex multiple mechanisms regulate human ST6GAL1 gene expression, including (a) alternative tissue-specific promoter usage (P1, P2, P3, and P4), (b) epigenetic mechanisms, and (c) gene copy number alteration [9,82]. Unlike many other cancers [69], CNV regulates ST6GAL1 mRNA levels in breast tumors. Aggressive TNBC tumors show higher rates of gene amplification than HR-positive breast tumors, suggesting that CNV is one mechanism linking higher ST6GAL1 levels in aggressive diseases, such as TNBC [12,27,70].

ST6GAL1 promoter DNA hypermethylation and silencing of ST6GAL1 expression have also been observed in multiple human cancers, including BCa [17,18,69,83]; however, the consequences of the ST6GAL1 signaling pathway in cancer pathobiology remain unclear. Control/adjacent normal breast tissue or normal breast epithelial cells have higher levels of ST6GAL1, which is primarily transcribed from the active, hypomethylated P3 promoter and, to a lesser extent, from the P2 promoter [9]. Our data analysis of primary breast tumors [14,84] reveals that the P3 and P2 promoters, but not the P1 or P4 promoters of human ST6GAL1, are highly methylated at the CpG islands. Hypermethylation of the ST6GAL1 P3 promoter, compared with the P2 promoter, is strongly associated with loss of ST6GAL1 in primary breast tumors, particularly in ER+ and HER2+ subtypes compared with the TNBC subtype, and, as expected, a hypomethylated promoter gives higher levels of ST6GAL1 expression in adjacent normal breast tissue controls. Our data suggest that P3 hypermethylation is the primary epigenetic switch responsible for silencing ST6GAL1 in primary BCa. ST6GAL1 as a marker of progressive disease, increased ST6GAL1 promoter methylation, and ST6GAL1 loss are associated with improved PFS in patients with BCa, suggesting a potential protective effect of primary tumor ST6GAL1 loss in certain BCa subtypes, such as ER+ or HER2+.

In contrast, ST6GAL1 is mostly dysregulated, predominantly transcriptionally activated in aggressive diseases [9,27,83,85]. Our results reveal that primary BCa, particularly aggressive HER2+ or TNBC subtypes, are sensitive to deletion of gene copy numbers associated with irreversible ST6GAL1 loss in BCa compared with normal/adjacent breast tissue controls. Epigenetic reactivation of cancer ST6GAL1 with the DNMT inhibitor (5-aza-dC) and the HDAC inhibitor entinostat (MS-275) reactivated endogenous cellular ST6GAL1 and stimulated the secretion of functional, exosome-bound exoST6 [27].

In cancer, profound heterogeneity in glycoprotein α2,6-sialylation mediated by ST6GAL1 is observed [15,16,27,32,48,64,86]. ST6GAL1, canonically thought to be natively expressed by cells, resides within the intracellular ER/Golgi secretory network, where it sialylates cell-autonomously nascent glycoproteins for display at the cell surface [13,14]. ST6GAL1 and other glycosyltransferases were also found in systemic circulation [87]. Generally, cells that express ST6GAL1 release the sialyltransferase into the extracellular milieu. While much of the circulating exoST6 originates from the liver, specifically from the P1 promoter of ST6GAL1, other host cells and cancer cells also contribute to the overall exoST6 pool [25,26,27,31,32]. Work from the Lau laboratory demonstrated that exoST6 can modify cell-surface glycans, even on cells that do not natively express ST6GAL1 [25,26,30,88,89,90,91], suggesting that the exoST6 axis contributes to the regulatory sialylation of cell-surface proteins. Changes in circulating exoST6 levels are associated with inflammatory states, other disease conditions, and malignancy [33,34,35,36,37,38,39]. Changes in circulating exoST6 levels are associated with inflammatory states, other disease conditions, and malignancy [33,34,35,36,37,38,39]. Elevated release of ST6 into the blood occurs during the hepatic acute-phase response [92]. A recent study found that serum ST6 may be a novel biomarker for highly malignant hepatocellular carcinoma (HCC). Patients with HCC and high serum ST6 levels exhibited more advanced disease and shorter RFS. However, it is not clear what the direct or indirect role of exoST6 is in malignancy [27,93]. Our earlier published results revealed that functional exoST6 can act on target TNBC cells that do not natively express ST6GAL1, enhancing TNBC growth, invasion, EMT, and cancer stemness ex vivo [27], confirming extracellular regulatory protein sialylation. The role of aberrant sialylation in promoting tumor cell survival, invasiveness, and therapeutic resistance has been demonstrated, primarily by altering key cell-surface receptors such as TNFR1, TGF-Rβ, and EGFR [13,85,94,95]. Our results from this manuscript further our understanding of sialylation heterogeneity in cancers by demonstrating epigenetic regulation and ST6GAL1 silencing in cancer, which may be one of the key contributors, in addition to the exoST6/sialation axis, to cancer heterogeneity in cell-surface α2-6 sialylation [27]. Our observation that higher DNA methylation of ST6GAL1 correlates with better PFS in BCa patients is striking, suggesting that loss of ST6GAL1 and reduced cell-surface sialylation may dampen the ST6GAL1/sialylation signaling axis in certain BCa subtypes, e.g., ER+ and HER2+.

In this manuscript, we have also investigated the stability of natively expressed human ST6GAL1 in cultured BCa cells. Cell-native ER/Golgi localized ST6GAL1 is an N-linked glycoprotein that is self-sialylated at Asn-146 and Asn-158 sites, and this self-sialylation is critical for human ST6GAL1’s structural stability [78,79]. As expected, sialidase treatment removes cellular protein/lipid-conjugated sialic acid [25] and structurally destabilizes and promotes proteasomal degradation [79] of endogenous ST6GAL1 protein levels in cultured BCa cells (Figure 8G).

While the ubiquitin–proteasome pathway actively turns over cellular ST6GAL1 [79], our results indicate that cellular ST6GAL1 in BCa cells is unstable; protein synthesis inhibitors (Cycloheximide, CHX) or proteasome inhibitors (MG132) degrade ST6GAL1 in multiple BCa cell lines (Figure 8G).

Our data analysis of DNA methylation at the CpG islands of ST6GAL1 promoters, linked to its loss of expression in human cancers [17,18,69,96], supports the hypothesis that reversible epigenetic changes, in addition to irreversible genomic copy number deletion in BCa, are key drivers of cancer heterogeneity and ST6GAL1-mediated α2-6 sialylation of cell-surface glycoproteins and signaling linked to cancer progression and metastasis [22,23,97]. Notably, our functional assays in cultured cells show that treatment with the DNMT inhibitor 5-aza-dC and the HDAC inhibitor MS-275 reverses epigenetic alterations and reactivates cellular ST6GAL1 expression and exoST6 in cultured BCa cell lines, confirming the causal role of methylation in gene silencing and highlighting the potential for pharmacologic intervention. Interestingly, epigenetic reactivation not only increases cellular ST6GAL1 and cell-surface α2,6 sialylation to promote BCa cell adhesion but also enhances the release of exosome-bound functional exoST6; exoST6 mediates the non-cell-autonomous sialylation of cell-surface glycoproteins, such as β-integrins. Ultimately, these findings highlight the epigenetic regulation of ST6GAL1 expression and the heterogeneity in cell-surface protein sialylation and exoST6 release as promising therapeutic targets for modulating tumor heterogeneity and cancer progression, warranting further study.

The clinical implications of these findings are significant. First, promoter methylation status of ST6GAL1 may serve as a predictive biomarker for disease progression and therapeutic response, particularly in ER+ and HER2+ BCa patients. Second, the reversibility of ST6GAL1 silencing with epigenetic drugs suggests new avenues for combination therapies to modulate the tumor glycome and overcome resistance mechanisms. Third, epigenetic drugs also release functional exoST6 associated with exosomes from BCa cells, suggesting that an exoST6/sialylation axis is key to remodeling target cells for therapeutic interventions in aggressive diseases, including TNBC.

However, the precise functional consequences of restoring ST6GAL1 expression and cell-surface protein sialylation—whether in terms of tumor growth, metastatic potential, or immune evasion—remain to be explored in both preclinical models and patient samples.

### There Are Several Limitations to Our Study

While we focused on ST6GAL1 promoter DNA methylation and ST6GAL1 expression in BCa, the downstream effects on global cell-surface glycoprotein α2,6 sialylation and specific receptor signaling were not systematically quantified in vivo. Additionally, the impact of other epigenetic modifications, such as histone modifications and non-coding RNAs, remains to be examined in detail in ex vivo and in vivo settings. Future work should also address the interplay between ST6GAL1 loss via DNA methylation and extracellular sialylation mediated by exoST6 in the context of the tumor immune microenvironment, given the known roles of glycosylation in immune recognition and evasion [26,91,98].

## 5. Conclusions

In summary, our results demonstrate that DNA methylation of the ST6GAL1 P3-promoter is a key determinant of ST6GAL1 expression loss and heterogeneity, in addition to ST6GAL1 copy number alterations, in human BCa. This epigenetic silencing is associated with a loss of cell-surface α2-6 sialylation and improved PFS in patients with ER+ and HER2+ BCa subtypes. Pharmacological reversal of DNA methylation can restore cellular ST6GAL1 expression and exoST6 associated with extracellular exosomal vesicles in cultured BCa cells, including TNBC cells, underscoring the dynamic and potentially targetable nature of this regulatory ST6GAL1/sialylation axis. Together, these findings establish the ST6GAL1/sialylation pathway as both a biomarker and a promising therapeutic target across BCa subtypes, including aggressive TNBC. Future studies should investigate the precise functional consequences of manipulating the intracellular ST6GAL1/sialylation axis vs. the exoST6/sialylation axis in vivo and explore ST6GAL1’s role in disease progression and response to therapy.

## Data Availability

Publicly available datasets were analyzed in this study.

## Supplementary Materials

The following supporting information can be downloaded at: https://www.mdpi.com/article/10.3390/cancers18182988/s1, Figure S1. Analysis of CpG content in the human P1, P2, and P3 promoters of ST6GAL1; Figure S2. Analysis of CpG content in the human ST6GAL2 promoters; Figure S3. Associations of ST6GAL1 mRNA expression with gene copy number variation (CNV) in BCa; Figure S4. Tumor-specific hypermethylation of the ST6GAL1 promoter, assessed using cg16751732 is associated with ST6GAL1 gene silencing and patient survival in an independent TCGA-BRCA dataset; Figure S5. ST6GAL1 promoter methylation at the primary site vs. lymph node (LN) metastatic site; Figure S6. Treatment-related cohorts were analyzed; Figure S7. Uncropped original Western blot images; Table S1. Subtype-Specific Promoter Methylation and Transcriptional Loss of ST6GAL1 Analysis of primary human breast tumor tissues revealed distinct epigenetic profiling between malignant and normal tissues; Table S2. Cg 15287850: BC patients’ clinicopathological features; Table S3. Cg 16751732: BC patients’ clinicopathological features; Table S4. Cg 25372568: BC patients’ clinicopathological features; Table S5. ST6GAL1 protein expression and BC patients’ clinicopathological features.
