# Supplementary material for: Epigenetic Regulation of Sialyltransferase ST6GAL1 Drives Breast Tumor Heterogeneity
Source: Cancers (Basel). 2026 Sep 16;18(18):2988. doi: 10.3390/cancers18182988 (PMC13605568; doi:10.3390/cancers18182988)
Supplement: Supplementary file 1 [file cancers-18-02988-s001.zip › cancers-4511468-supplementary.pdf]

ST6GAL1

NM_173216, NM_73217

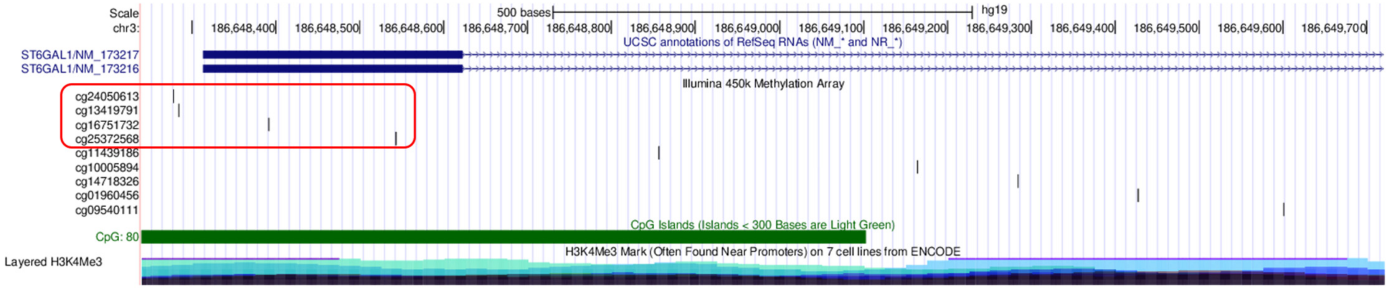

NM_003032

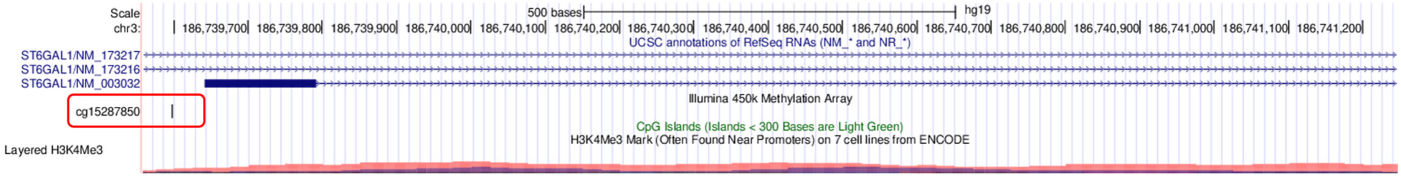

Figure S1. Analysis of CpG content in the human P1, P2, and P3 promoters of *ST6GAL1*. Genomic positions of the ST6GAL1 gene and its exon structures were obtained from the Ensembl genome browser (http://www.ensembl.org). Genomic positions and annotations for methylation probes were obtained using the Bioconductor annotation package "IlluminaHumanMethylation450kanno.ilmn12.hg19". The Figure was generated using the UCSC Genome Browser (https://genome.ucsc.edu). All genomic coordinates were based on the hg19 reference genome.

ST6GAL2

https://genome.ucsc.edu/cgi-bin/hgTracks?db=hg19&lastVirtModeType=default&lastVirtModeExtraState=&virtModeType=default&virtMode=0&nonVirtPosition=&position=chr2%3A107426606%2D107512114&hgsid=3972755811_Fg5azqbYaRl2gXotC8j1nzIjZz2L

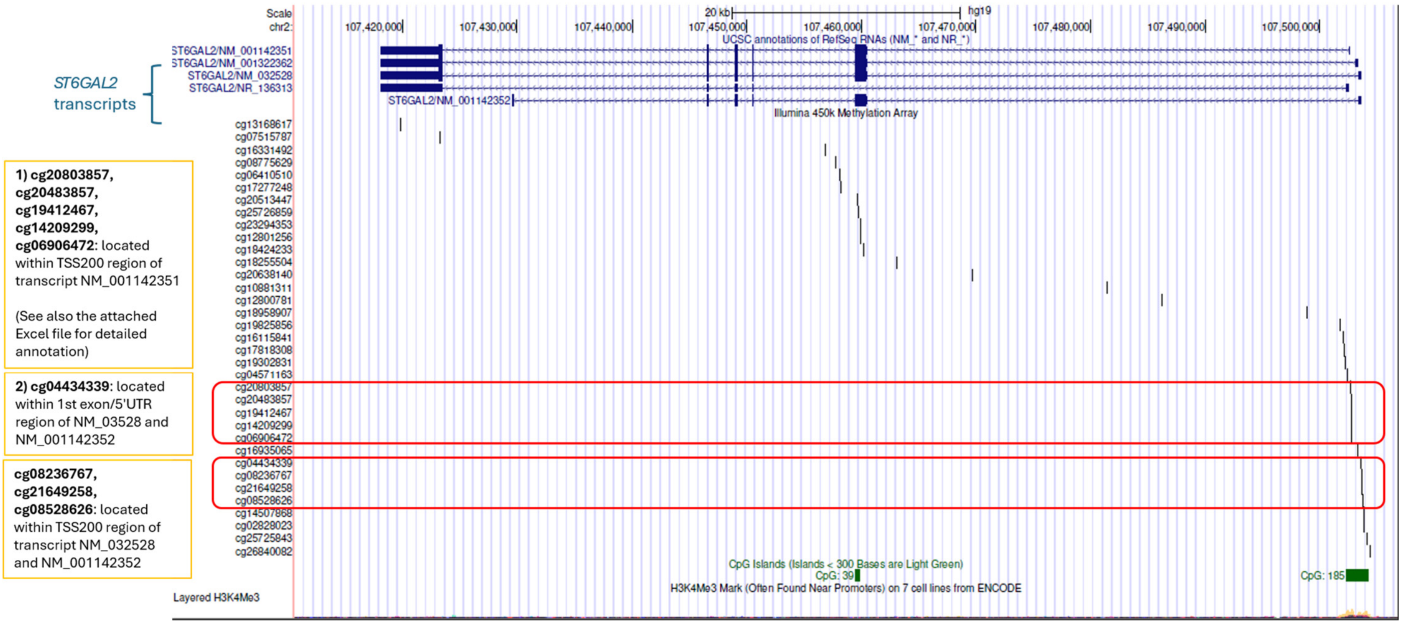

Figure S2. Analysis of CpG content in the human ST6GAL2 promoters. Genomic positions of the ST6GAL2 gene and exon structures were obtained from the Ensembl genome browser (http://www.ensembl.org). Genomic positions and annotations of methylation probes were obtained using the Bioconductor annotation package "IlluminaHumanMethylation450kanno.ilmn12.hg19". The Figure was generated using the UCSC Genome Browser (https://genome.ucsc.edu). All genomic coordinates were based on the hg19 reference genome.

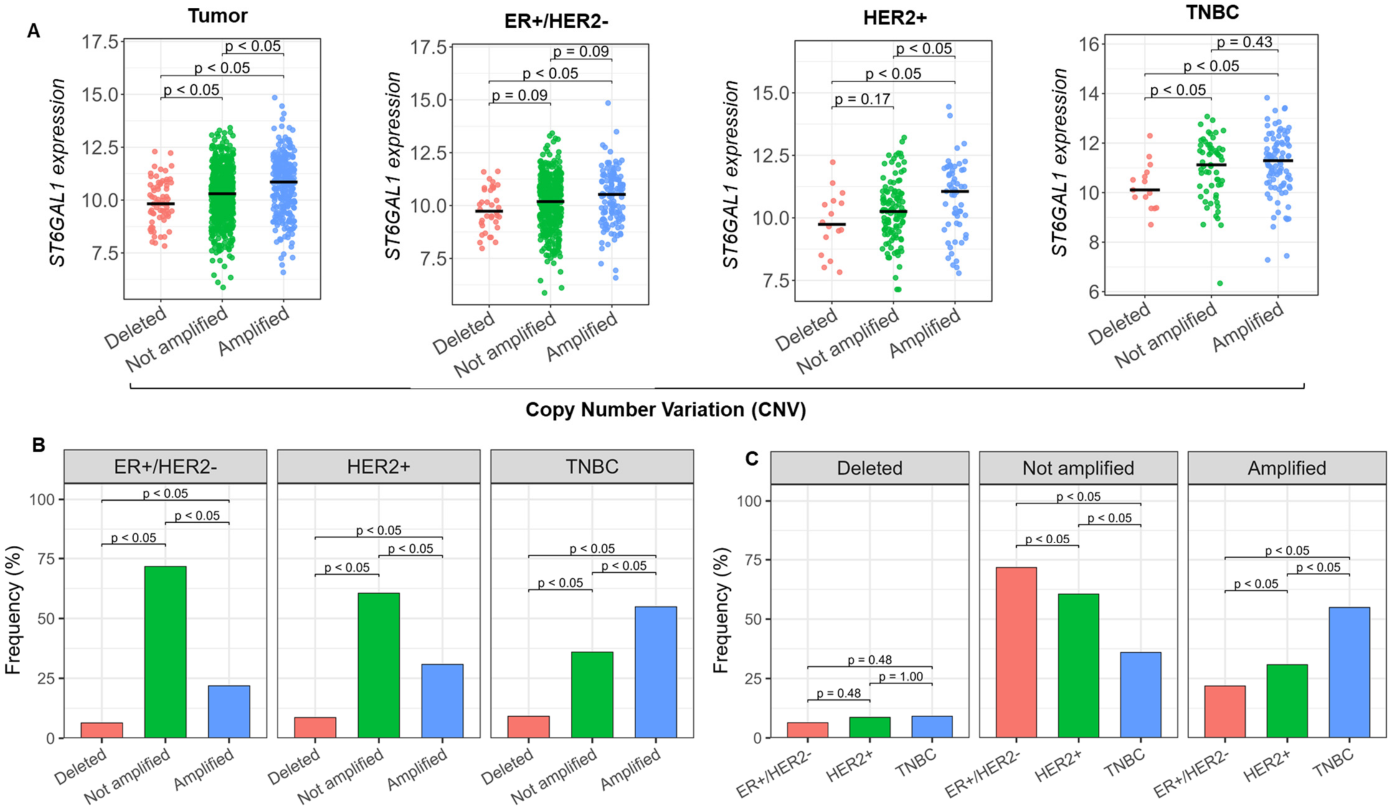

Figure S3. Associations of ST6GAL1 mRNA expression with gene copy number variation (CNV) in BCa. (**A-B**) ST6GAL1 copy number variation and its frequency (%) and mRNA expression data from the TCGA BRCA cohort were analyzed by stratifying tumor samples into deletion, not-amplified, and amplified groups. Total breast tumors (deleted, n = 67, neutral, n = 574, and amplified, n = 270), ER+/HER2- tumors (deleted, n = 36, neutral, n = 403, and amplified, n = 123), HER2+ tumors (deleted, n = 16, neutral, n = 112, and amplified, n = 57), and TNBC tumors (deleted, n = 15, neutral, n = 59, and amplified, n = 90) were analyzed in the TCGA-BRCA database (total patients: n = 1045; Normal: n = 134; ER+/HER2-: n = 562; HER2+: n = 185; TNBC: n = 164). Differences in ST6GAL1 expression were assessed using Kruskal–Wallis tests, with post hoc comparisons and $p < 0.05$ defining statistical significance in R.

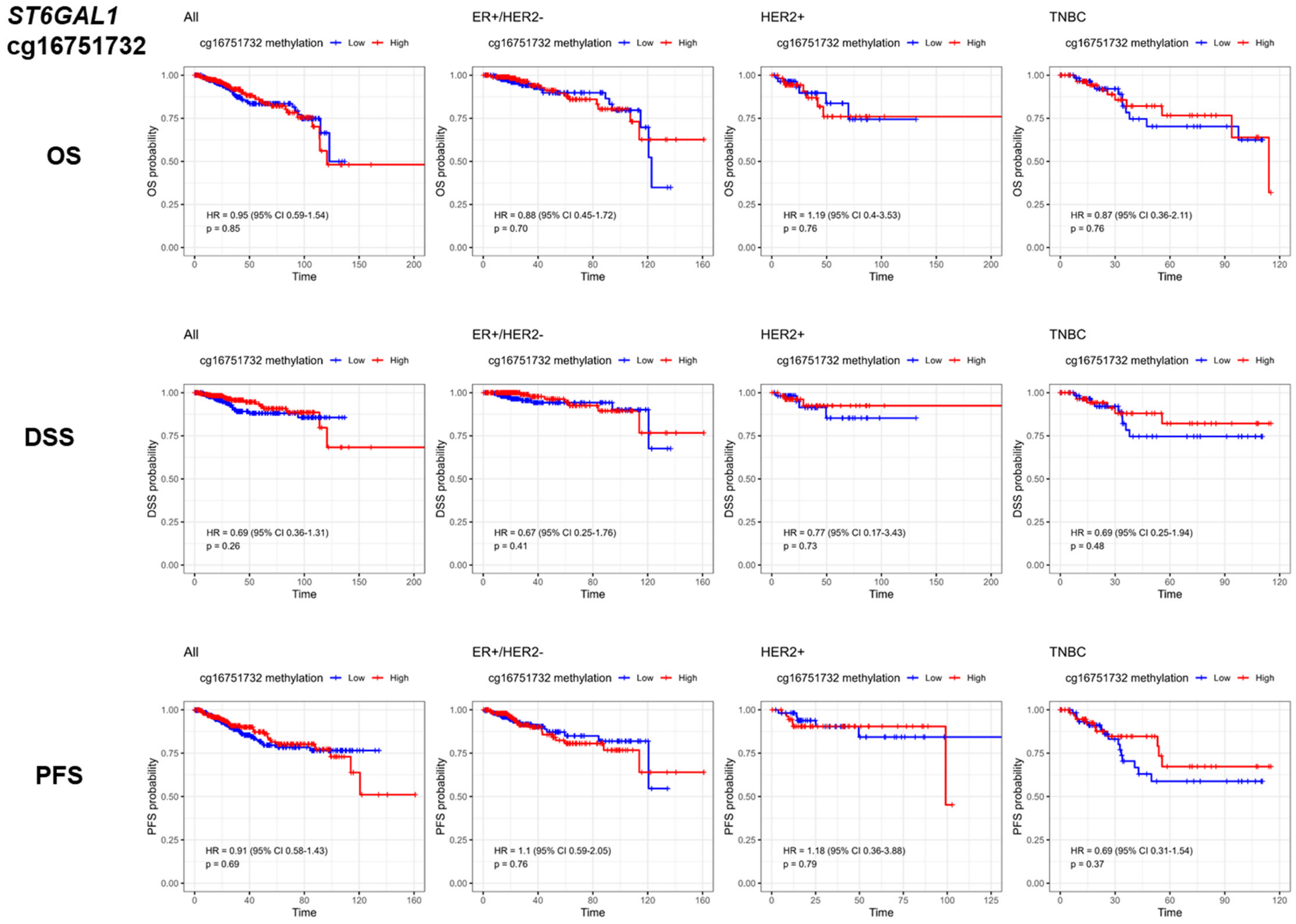

Figure S4. Tumor-specific hypermethylation of the ST6GAL1 promoter, assessed using cg16751732 is associated with ST6GAL1 gene silencing and patient survival in an independent TCGA-BRCA dataset. Methylation data were analyzed using β-values. For comparisons of patient survival outcomes, cases were dichotomized into high- and low-methylation groups based on the median methylation value for each probe.

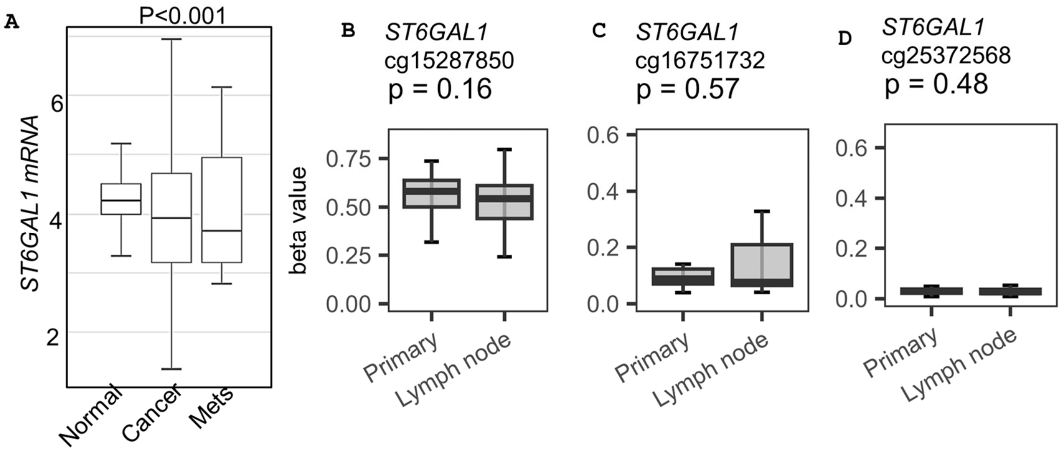

Figure S5. ST6GAL1 promoter methylation at the primary site vs. lymph node (LN) metastatic site. (**A**) TCGA-BRCA dataset: ST6GAL1 mRNA expression in normal breast (n=113), BC patients (n=1275), and metastatic patients (n=7). Mann–Whitney U test, p<0.0001. Analysis performed on the UCSC Xena platform. (**B-D**) Metastatic cohorts were analyzed using GSE58999 (n = 44), obtained from the Gene Expression Omnibus (GEO) via the GEOquery package in R. See Methods section.

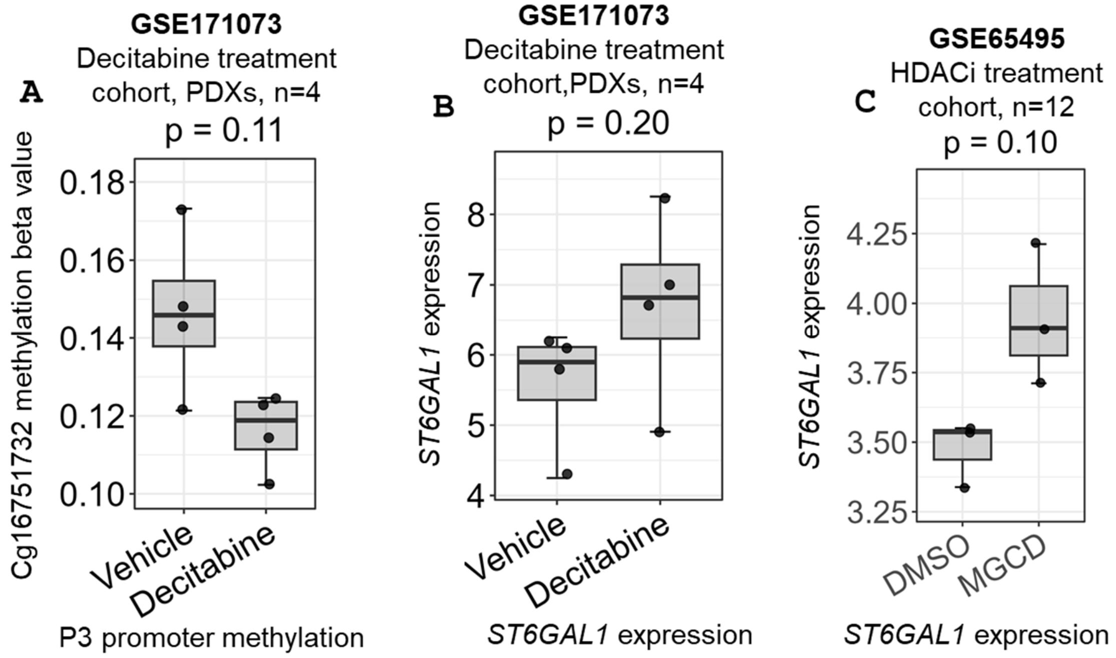

Figure S6. Treatment-related cohorts were analyzed using (**A-B**) GSE171073 (n=4 per group, PDXs) and (**C**) GSE65495 (n=12 per group, BC patients), obtained from the Gene Expression Omnibus (GEO) via the GEOquery package in R. See the Methods section.

Supplementary Table S1: Subtype-Specific Promoter Methylation and Transcriptional Loss of *ST6GAL1*
Analysis of primary human breast tumor tissues revealed distinct epigenetic profiling between malignant and normal tissues. Notably, the methylation status varied significantly by promoter locus and molecular subtype:

**Tissue type/subtype**	**P2-promoter methylation (cg15287850, chr3:186,739,599; −43 bp)**	**Patient Survival: cg cg15287850**	**P3-promoter methylation (cg16751732, chr3:186,648,392; +78 bp)**	**Patient Survival: cg cg16751732**	**P3-promoter methylation (cg25372568, chr3: 186,648,544; +230 bp)**	**Patient Survival: cg cg25372568**
**Adjacent normal breast tissue**	High vs. low mRNA (R=-0.25, p=0.023, vs. mRNA)	-	Low vs. high mRNA (R=0.23, p<0.05)	-	Unchanged vs. mRNA (n.s.)	-
**ER+ BC subtype**	Low vs. high mRNA (R=0.29, p<0.001)	OS: Good, trend (HR = 0.54, p=0.08, n.s.); PFS: Good, (HR = 0.44, p<0.05)	High vs. low mRNA (R=-0.28, p<0.001)	n.s.	High vs. low mRNA (R=-0.26, p<0.001)	OS: Good, trend (HR = 0.35, p<0.05)
**HER2+ BC subtype**	Unchanged vs. mRNA (n.s.)	OS: Good (HR = 0.15, p<0.05; DSS: Good, trend (HR = 0.13, p=0.06, n.s.); PFS: Good (HR = 0.19, p<0.05)	High vs. low mRNA (R=0.45, p<0.001)	n.s.	High vs. low mRNA (R=-0.44, p<0.001)	n.s.
**TNBC subtype**	Unchanged vs. mRNA (n.s.)	n.s.	Unchanged vs. mRNA (n.s.)	n.s.	Unchanged vs. mRNA (n.s.)	n.s.

Values represent median DNA methylation β-values and median *ST6GAL1* mRNA expression levels (TPM values). Methylation or expression levels are relative to normal within each subtype. P-values were calculated using the Mann–Whitney U test comparing each breast cancer subtype with normal breast tissue.

Supplementary Table S2: Cg15287850: BC patients clinicopathological features

Clinical factor	Low<br>N = 328[1]	High<br>N = 329[1]	p-value[2]
AGE	59 [29, 90]	57 [26, 90]	0.072
SEX			0.3
Female	318 (98%)	325 (99%)	
Male	6 (1.9%)	2 (0.6%)	
menopause			0.4
Post	223 (74%)	214 (71%)	
Pre	77 (26%)	88 (29%)	
RACE			0.021
Asian	18 (5.7%)	13 (4.0%)	
Black	81 (26%)	55 (17%)	
other	0 (0%)	1 (0.3%)	
White	215 (68%)	253 (79%)	
IHC_SUBTYPE2			0.10
ER+/HER2-	197 (60%)	219 (67%)	
HER2+	67 (20%)	47 (14%)	
TNBC	64 (20%)	63 (19%)	
GRADE			0.002
1	60 (19%)	77 (25%)	
2	117 (38%)	138 (46%)	
3	131 (43%)	88 (29%)	
T_stage			0.6
T1	76 (24%)	91 (28%)	
T2	193 (60%)	182 (56%)	
T3	46 (14%)	47 (14%)	
T4	8 (2.5%)	7 (2.1%)	
N_stage			0.8
N0	146 (46%)	146 (45%)	
N1	104 (33%)	112 (34%)	
N2	43 (14%)	38 (12%)	
N3	23 (7.3%)	29 (8.9%)	
M_stage			0.079
M0	253 (97%)	254 (100%)	
M1	7 (2.7%)	1 (0.4%)	

[1]Median [Min, Max]; n (%)

[2]Wilcoxon rank sum test; Pearson's Chi-squared test

Supplementary Table S3: Cg16751732: BC patients clinicopathological features

Clinical factor	Low<br>N = 328[1]	High<br>N = 328[1]	p-value[2]
AGE	58 [26, 90]	58 [28, 90]	0.5
SEX			0.7
Female	323 (98%)	319 (99%)	
Male	5 (1.5%)	3 (0.9%)	
menopause			0.003
Post	238 (78%)	198 (67%)	
Pre	67 (22%)	98 (33%)	
RACE			0.8
Asian	16 (5.0%)	15 (4.8%)	
Black	70 (22%)	66 (21%)	
other	1 (0.3%)	0 (0%)	
White	235 (73%)	232 (74%)	
IHC_SUBTYPE2			0.008
ER+/HER2-	198 (60%)	217 (66%)	
HER2+	51 (16%)	63 (19%)	
TNBC	79 (24%)	48 (15%)	
GRADE			0.3
1	60 (20%)	77 (25%)	
2	130 (43%)	124 (40%)	
3	112 (37%)	107 (35%)	
T_stage			0.9
T1	88 (27%)	78 (24%)	
T2	187 (57%)	188 (59%)	
T3	46 (14%)	47 (15%)	
T4	7 (2.1%)	8 (2.5%)	
N_stage			0.2
N0	157 (48%)	134 (43%)	
N1	111 (34%)	105 (33%)	
N2	35 (11%)	46 (15%)	
N3	22 (6.8%)	30 (9.5%)	
M_stage			0.7
M0	254 (98%)	252 (99%)	
M1	5 (1.9%)	3 (1.2%)	

[1]Median [Min, Max]; n (%)

[2]Wilcoxon rank sum test; Pearson's Chi-squared test

Supplementary Table S4: Cg25372568: BC patients clinicopathological features

Clinical factor	Low N = 328[1]	High N = 329[1]	p-value[2]
AGE	56 [27, 90]	59 [26, 90]	0.2
SEX			0.3
Female	323 (99%)	320 (98%)	
Male	2 (0.6%)	6 (1.8%)	
menopause			0.2
Post	209 (70%)	228 (75%)	
Pre	90 (30%)	75 (25%)	
RACE			0.053
Asian	14 (4.4%)	17 (5.3%)	
Black	55 (17%)	81 (25%)	
other	1 (0.3%)	0 (0%)	
White	247 (78%)	221 (69%)	
IHC_SUBTYPE2			0.015
ER+/HER2-	197 (60%)	219 (67%)	
HER2+	53 (16%)	61 (19%)	
TNBC	78 (24%)	49 (15%)	
GRADE			>0.9
1	67 (22%)	70 (23%)	
2	128 (42%)	127 (41%)	
3	107 (35%)	112 (36%)	
T_stage			0.5
T1	91 (28%)	76 (23%)	
T2	185 (57%)	190 (58%)	
T3	43 (13%)	50 (15%)	
T4	6 (1.8%)	9 (2.8%)	
N_stage			0.4
N0	137 (43%)	155 (48%)	
N1	112 (35%)	104 (33%)	
N2	46 (14%)	35 (11%)	
N3	26 (8.1%)	26 (8.1%)	
M_stage			0.3
M0	256 (98%)	251 (99%)	
M1	6 (2.3%)	2 (0.8%)	

[1]Median [Min, Max]; n (%)
[2]Wilcoxon rank sum test; Pearson's Chi-squared test

Supplementary Table S5: ST6GAL1 protein expression and BC patients' clinicopathological features

Clinical factor	Low N = 328[1]	High N = 329[1]	p-value[2]
AGE	57 [26, 90]	59 [28, 90]	0.13
SEX			0.076
Female	319 (98%)	324 (100%)	
Male	7 (2.1%)	1 (0.3%)	
menopause			0.012
Post	202 (68%)	235 (77%)	
Pre	96 (32%)	69 (23%)	
RACE			0.5
Asian	13 (4.1%)	18 (5.6%)	
Black	72 (23%)	64 (20%)	
other	0 (0%)	1 (0.3%)	
White	231 (73%)	237 (74%)	
IHC_SUBTYPE2			0.005
ER+/HER2-	223 (68%)	193 (59%)	
HER2+	58 (18%)	56 (17%)	
TNBC	47 (14%)	80 (24%)	
GRADE			0.4
1	63 (21%)	74 (24%)	
2	134 (44%)	121 (39%)	
3	106 (35%)	113 (37%)	
T_stage			0.3
T1	75 (23%)	92 (28%)	
T2	190 (58%)	185 (57%)	
T3	53 (16%)	40 (12%)	
T4	7 (2.2%)	8 (2.5%)	
N_stage			0.5
N0	136 (43%)	156 (48%)	
N1	111 (35%)	105 (33%)	
N2	43 (13%)	38 (12%)	
N3	29 (9.1%)	23 (7.1%)	
M_stage			0.3
M0	252 (99%)	255 (98%)	
M1	2 (0.8%)	6 (2.3%)	

[1]Median [Min, Max]; n (%)
[2]Wilcoxon rank sum test; Pearson's Chi-squared test

Figure S7. Uncropped original Western blot images. To ensure absolute accuracy and proper labeling when cropping Western blot bands from our full-sized images, we implemented the following steps of verification protocol: We loaded a Bio-Rad pre-stained protein ladder alongside our samples in every gel. Because the ladder contains distinct, colored reference bands (known molecular weights) that remain visible on the membrane throughout the transfer process, after protein transfer, we marked the protein ladders with a pencil for better visibility during blot processing. When cropping the target band region, we kept the adjacent ladder lane in the frame or cropped it in exact parallel alignment with the sample lanes. This allowed us to continuously cross-reference the target protein's molecular weight against the known standard sizes of the Bio-Rad ladder, eliminating any risk of misidentification or lane shifting. We always optimized Western blots with specific antibodies to ensure clear, single-band detection at the expected molecular weight.

Supplementary Figure S7

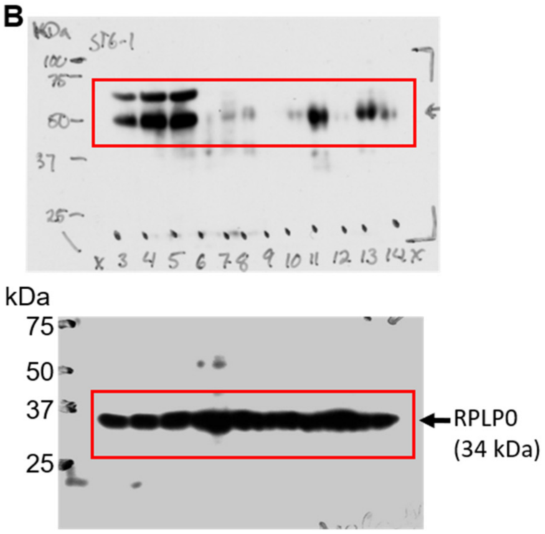

Figure 7

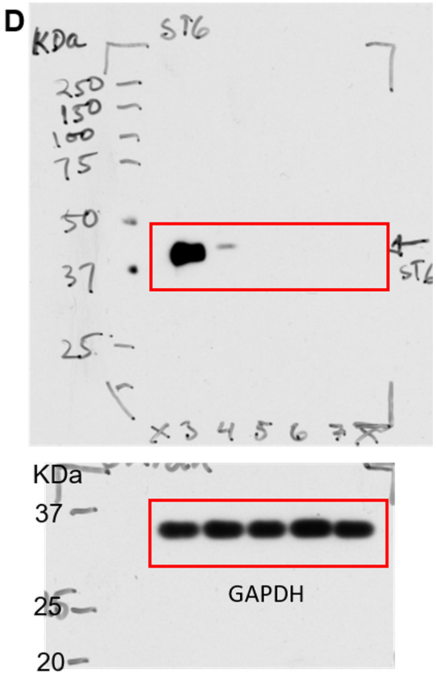

Figure 7

Supplementary Figure S7

Supplementary Figure S7

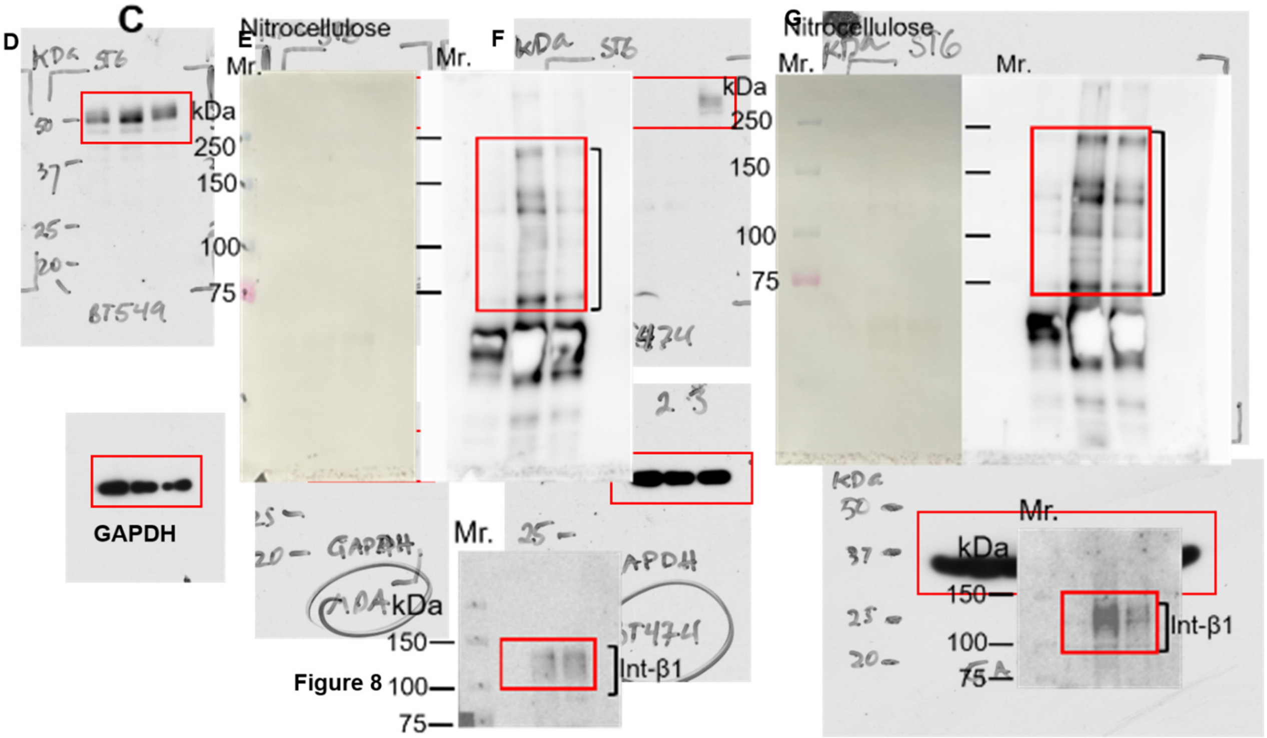

Figure 8

Figure 9

Supplementary Figure S7

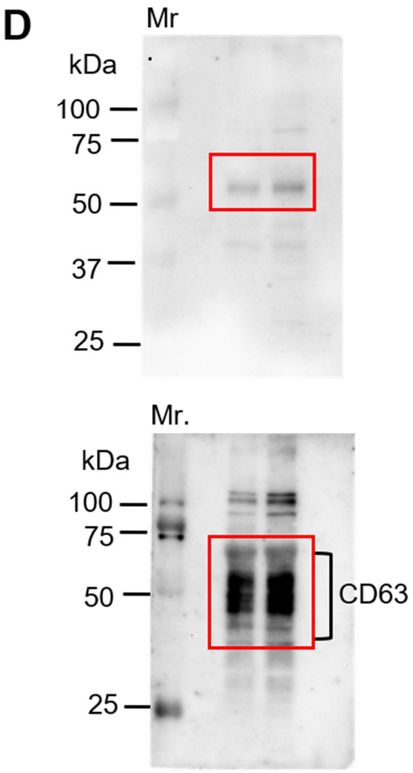

Figure 9

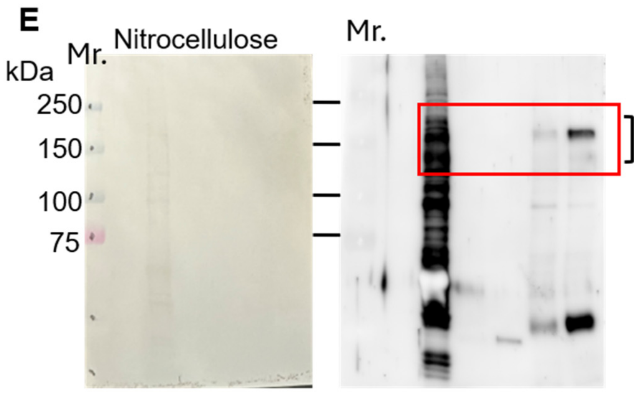

Figure 9
